# Accurate Computational Prediction of Core-Electron Binding Energies in Carbon-Based Materials: A Machine-Learning Model Combining Density-Functional Theory and *GW*

**DOI:** 10.1021/acs.chemmater.1c04279

**Published:** 2022-07-13

**Authors:** Dorothea Golze, Markus Hirvensalo, Patricia Hernández-León, Anja Aarva, Jarkko Etula, Toma Susi, Patrick Rinke, Tomi Laurila, Miguel A. Caro

**Affiliations:** † Faculty of Chemistry and Food Chemistry, Technische Universität Dresden, 01062 Dresden, Germany; ‡ Department of Applied Physics, Aalto University, 02150 Espoo, Finland; § Department of Electrical Engineering and Automation, Aalto University, 02150 Espoo, Finland; ∥ Department of Chemistry and Materials Science, Aalto University, 02150 Espoo, Finland; ⊥ University of Vienna, Faculty of Physics, Boltzmanngasse 5, 1090 Vienna, Austria

## Abstract

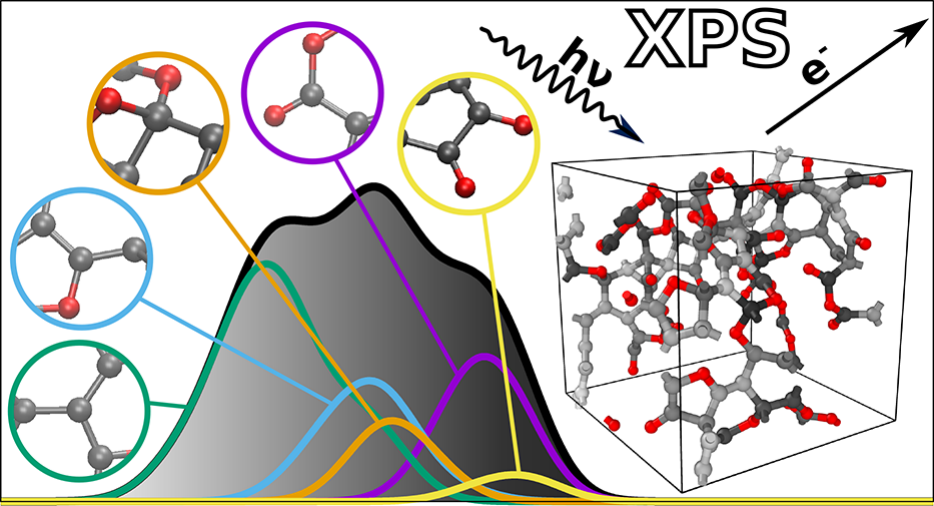

We present a quantitatively accurate machine-learning (ML) model for the computational prediction of core–electron binding energies, from which X-ray photoelectron spectroscopy (XPS) spectra can be readily obtained. Our model combines density functional theory (DFT) with *GW* and uses kernel ridge regression for the ML predictions. We apply the new approach to disordered materials and small molecules containing carbon, hydrogen, and oxygen and obtain qualitative and quantitative agreement with experiment, resolving spectral features within 0.1 eV of reference experimental spectra. The method only requires the user to provide a structural model for the material under study to obtain an XPS prediction within seconds. Our new tool is freely available online through the XPS Prediction Server.

## 1 Introduction

X-ray spectroscopy techniques are routinely used for structural characterization of materials^1^ and molecules.^2^ Among the different X-ray spectroscopies, X-ray photoelectron spectroscopy (XPS) is arguably the most widespread. In XPS, a material sample is irradiated with monochromatic X-rays to probe the binding energy (BE) of its core electrons. When a core electron absorbs an X-ray photon with enough energy, it leaves the sample and its kinetic energy can be measured. Since the X-ray incident energy is known, the difference between the kinetic and the incident energy is the core–electron BE. This energy is characteristic of the chemical environment of the core-excited atom. XPS spectra are therefore frequently used for structural characterization.^2,3^

Carbon-based materials, such as amorphous and disordered carbons, graphene, graphene derivatives, and nanotubes are an important material class in industry and research.^4−6^ Furthermore, emerging applications are envisioned, such as energy storage and conversion,^7^ electronics,^8^ electrocatalysis,^9,10^ and biosensing.^11^ Unfortunately, the atomic structure of carbon-based materials is often not completely known because, in addition to their possibly disordered nature, they also often contain a wide variety of defects and surface chemical functionalizations. XPS is one of the most commonly used spectroscopy tools for structural characterization of carbon-based materials.^11,12^ However, it is generally difficult and sometimes impossible to establish the precise origin of each peak in an XPS spectrum due to the lack of well-defined reference data.^13^ In addition, the link between atomic structure and a particular XPS spectrum is often imprecise since the core–electron BEs of two atoms in different chemical environments can be the same. Further ambiguities are introduced by the peak fitting procedure, which must be applied to resolve overlapping features in the experimental spectrum and which often relies on a number of assumptions, such as the total number of peaks.^13,14^ These limitations impact the interpretation of experimental XPS spectra. Inferring the atomic structure from the XPS spectrum is referred to as the “backward” route. An alternative strategy is to generate XPS spectra from candidate structural models. The best match between generated and measured spectra then provides the best structural model. We call this the “forward” direction.

For the forward route to be feasible, we require a pool of candidate structures and a theoretical or computational approach that is able to accurately predict core–electron BEs from the atomic structure alone. Candidate structures can be generated computationally. The difficulty with the forward direction therefore lies with the availability and computational cost of accurate XPS prediction tools. Currently, XPS modeling is almost exclusively based on Kohn–Sham density functional theory (DFT) employing so-called Δ-methods, such as the Delta self-consistent field (ΔSCF)^15^ or Delta Kohn–Sham (ΔKS) frameworks.^16^ DFT is, by design, a ground-state theory and does not provide systematic access to excited-state properties. However, for small molecules, relative and absolute core-level BEs from ΔSCF and ΔKS generally compare well to experiment, in particular when employing meta-generalized gradient approximation (meta-GGA) functionals.^17−20^ Recently, ΔSCF calculations with meta-GGAs were, in combination with finite-size correction schemes, also successfully applied to simple solids.^21^

While the functional dependence of molecular core-level excitations is moderate for absolute^22,23^ and even negligible for relative BEs,^17^ it can be more severe for complex materials.^24−26^ As a consequence of the self-interaction error, the accuracy of DFT-based Δ-methods deteriorates with increasing system sizes, which has been comprehensively discussed for valence excitations^27^ and has been also observed for core states.^28^ The *GW* approximation^29^ to many-body perturbation theory overcomes these limitations of DFT and provides a rigorous quantum-mechanical framework for the photoemission process,^30^ see also refs (28) and (63) for a discussion of the limitations of Δ-methods. However, the computational cost of a *GW* calculation is orders of magnitude larger than for DFT, making a straightforward application of *GW* to complex materials difficult.

The computational prediction of XPS spectra of amorphous structures requires sampling over all atoms in the structure, which is demanding even with DFT and impossible with *GW*. Machine-learning (ML) methods are a promising strategy to bridge the gap between high accuracy and computational efficiency. While the application of ML in spectroscopy is still in its infancy,^31−34^ the first proof-of-concept applications for the prediction of valence^35^ and core-level spectra are emerging.^36−38^ In this paper, we advance these ideas for real-world applications and develop a powerful XPS prediction tool by combining ΔKS calculations and highly accurate *GW* predictions with ML models. We show that our method can provide access to quantitatively accurate predictions of XPS spectra of complex disordered materials and small molecules containing carbon, hydrogen, and oxygen (CHO).

The remainder of this article is structured as follows: We discuss the generation of structural models for CHO-containing compounds in section 2.1. We proceed with the details of the electronic-structure methods used to generate computational XPS data in section 2.2. The architecture of our ML models is discussed in section 2.3. The performance of the ML models for C 1s and O 1s excitations of molecular and extended CHO structures is presented in section 3, followed by XPS spectra predictions for selected CHO materials. Finally, we introduce our XPS Prediction Server as a freely available online tool and draw conclusions in section 4.

## 2 Methods

### 2.1 Structural Models of CHO-Containing Compounds

Due to carbon’s versatility in chemical bond formation, the composition and configuration space for carbon-based materials and molecules is vast.^39,40^ Diverse examples of CHO compounds are small molecules (water, methane, methanol, etc.), large molecules and polymers (lipids, sugars, cellulose, etc.), and solid-state materials. The main focus of this work are CHO materials. While two distinct CHO materials will differ from each other when regarded as a whole, they are made of the same (or very similar) individual building blocks, or atomic motifs. By building a library of structural models for CHO materials, we can identify the individual atomic motifs most representative of the ensemble.^41^ Effective motif selection, discussed in more detail in section 2.1.2, is essential to obtain a compact and manageable representation of large structural databases.

#### 2.1.1 CHO Structural Databases

We have generated two CHO structure databases for the prediction of XPS data: one for CHO materials and another one for small CHO-containing molecules. The structural database for CHO materials was constructed from computer-generated model structures of amorphous carbon (a-C), hydrogenated a-C (a-C:H), oxygen-enriched a-C (a-C:O), functionalized a-C, oxygenated amorphous carbon (a-CO_*x*_), graphene (G), and reduced graphene oxide (rGO). All of the computational structural models for these materials are taken from the available literature.^41−47^ An exception are the oxygen-rich a-CO_*x*_ models, which were generated using DFT molecular dynamics following ref (47). The CHO materials in our structural database cover a broad range of structural building blocks, which provides the necessary foundation to map all the characteristic atomic motifs centered on O and C (H lacks an atomic core) to their corresponding core–electron binding energies.

Our structural database of small CHO molecules is a subset of the QM9 data set,^48^ which contains in total 134k organic compounds. Our QM9 subset consists of 2089 CHO molecules with 3–29 atoms and its size distribution follows that of those QM9 molecules which contain exclusively C, H, and O. The full QM9 database also includes molecules with N and F. Those molecules were not considered for the subset generation. The molecules in our subset contain up to 9 “heavy” atoms (in this case C or O), which amounts to a total of 14 707 C 1s and 1 865 O 1s excitations. We found that the relationship between local molecular structure and XPS properties is not transferable to extended CHO structures, i.e., these data were not used for the generation of the ML models for CHO materials. However, since these molecules are small, calculating their XPS spectra is computationally comparatively inexpensive. This allows us to both benchmark our methodology in the limit of abundant data and produce a useful reference database of computational XPS spectra of small CHO-containing molecules, which is valuable on its own.

#### 2.1.2 Structure Classification Based on Representative Atomic Motifs

The computational structures for the CHO materials contain many C and O sites. Computing the 1s core–electron BE for each site is, unlike for molecules, computationally too expensive, in particular at the *GW* level of theory. Therefore, to make the computational effort tractable, we have identified the most representative atomic motifs in the entire CHO materials database, using the data clustering methodology presented in ref (41). In short, a many-body atomic descriptor known as the “smooth overlap of atomic positions” (SOAP) is used to encode the atomic structure surrounding each atomic site in the database of structures.^49^ These descriptors allow us to construct kernel functions, which can also be understood as measures of similarity between the atomic environments. From these, we can build a distance matrix that can then be fed to a data classification algorithm. This algorithm, *k*-medoids in our case,^50^ clusters data (here the atomic environments) into groups that share similarities and assigns a “medoid” (also called a “centroid” in barycenter-based clustering methods). This medoid corresponds to the most representative atomic motif within each data cluster. We can preselect the number of data clusters to build, based on our estimate of available CPU power, and perform the core–electron calculations only on those. This leads to efficient charting of configuration space, since we avoid repeatedly calculating motifs that are overrepresented in the database of atomic structures.

**Figure 1:**
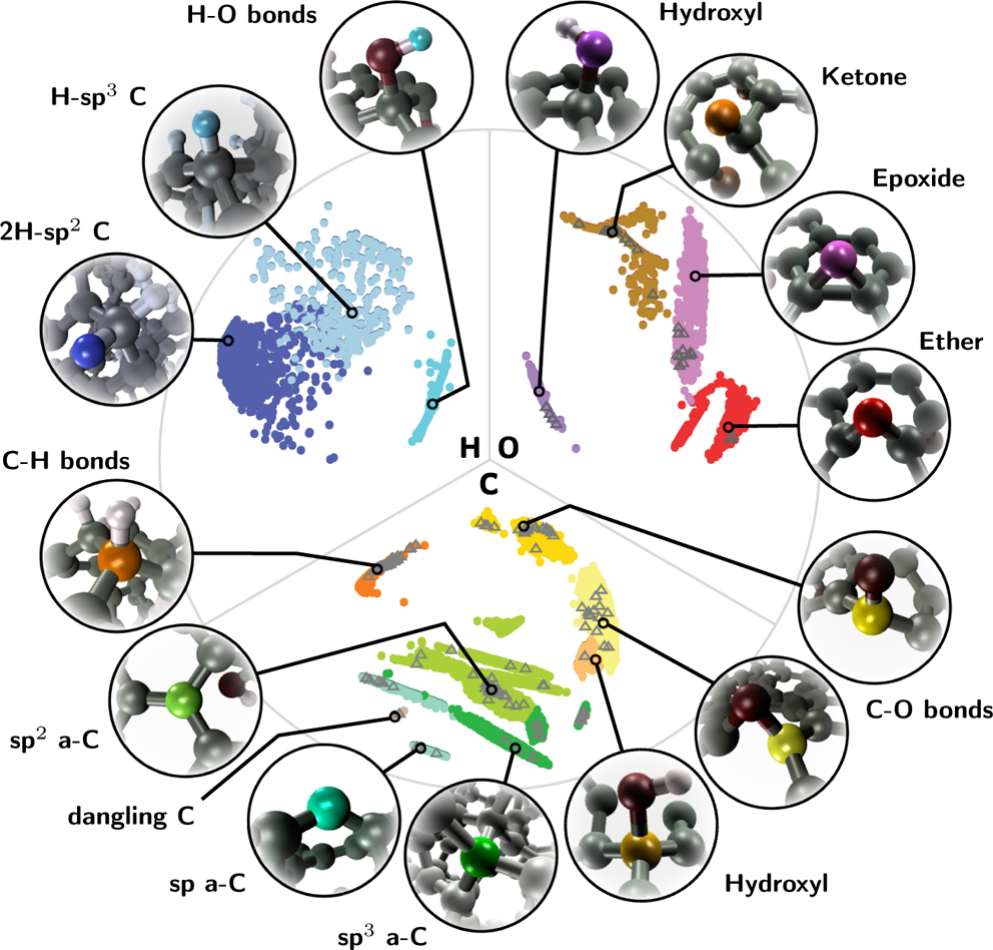
Cluster-based multidimensional scaling map of the CHO materials database used in this work. The graph is partitioned into three sections, depending on whether a C, H, or O atom is at the center of the environment. For instance, a hydroxyl group is viewed differently depending on whether the atomic environment descriptor uses C, H, or O as the origin. The distance between data points on the map is inversely proportional to the degree of similarity between the corresponding atomic environments. This similarity is established using SOAP many-body descriptors,^49,51^ as explained in more detail in refs (41, 52, and 53). The gray triangles indicate the motifs selected for the *GW* calculations.

The composition of the database is visualized in Figure 1, where we show a map of chemical and structural similarities between the present atomic motifs using a low-dimensional embedding tool, cl-MDS,^54^ which combines ML atomic descriptors^49,51^ and multidimensional scaling (a dimensionality reduction technique)^41,52,53^ with data clustering.^50^ Here, an atomic motif is constructed from a central C, H, or O atom embedded within diverse CHO environments. Atomic motifs are associated in classical terms with coordination environments (e.g., sp, sp^2^, and sp^3^ in carbon^41^) or with chemical groups, such as keto, epoxide, hydroxyl groups, etc. The central atom is either part of this group or adjacent to it, see Figure 1. The environment within the immediate vicinity of the central atom includes all the neighbor atoms within a given cutoff sphere. To simplify visualization, we used a cutoff radius of ∼2.25 Å to generate the similarities in Figure 1. However, this radius is too small when selecting structures for training the ML models. Motivated by the convergence studies for finite systems discussed in section 2.1.3, we retain structural information within a cutoff sphere of radius 4.25 Å for all the ML models trained in this study.

The subset of atomic motifs in the database selected for *GW* calculations is indicated with gray triangles in Figure 1. ΔKS calculations are performed on an extended subset of the whole database that includes the *GW* environments. Note that core-level calculations are only performed for heavy atoms, and we include thus only motifs with central C or O atom. Using the structures selected for *GW* as an example (the gray triangles), Figure 1 visually highlights two aspects of motif selection: (i) The diversity of our CHO database is preserved in the selection of those structures used for ML model construction, i.e., we draw samples from all over the map. (ii) This selection needs to be carried out according to the longer cutoff, since a classification based on a shorter cutoff, while useful in visualization, does not contain enough information to train a predictive ML model (the drawn samples are not homogeneously distributed on the map). If we drew the same map according to similarities based on the 4.25 Å cutoff, Figure 1 would look very different and it would be impossible to make an intuitive connection between the map and classical chemical group classification. See Figure S5 of the Supporting Information for a cl-MDS map with the larger cutoff.

#### 2.1.3 Carving out the Structures

Another issue pertains to the size of the input structures, which are model “supercells” with up to 355 atoms with periodic boundary conditions. The cost of DFT calculations scales cubically with the number of atoms in the system. The cost of core-level *GW* calculations is even more formidable and scales with the fifth power of the number of atoms.^28^ We therefore resort to moderately sized cluster models of around 100 atoms for our carbon structures. Cluster models are justified for core-level excitations, since local atomic properties, such as the core–electron binding energy, are expected to converge with respect to the number of neighbors explicitly included in the calculation. By only keeping the atoms in the immediate surrounding of the core-excited atom, we can significantly speed up the core-level calculations. At the same time, we remove the need for periodic boundary conditions, which are currently incompatible with our core-level *GW* implementation.

**Figure 2:**
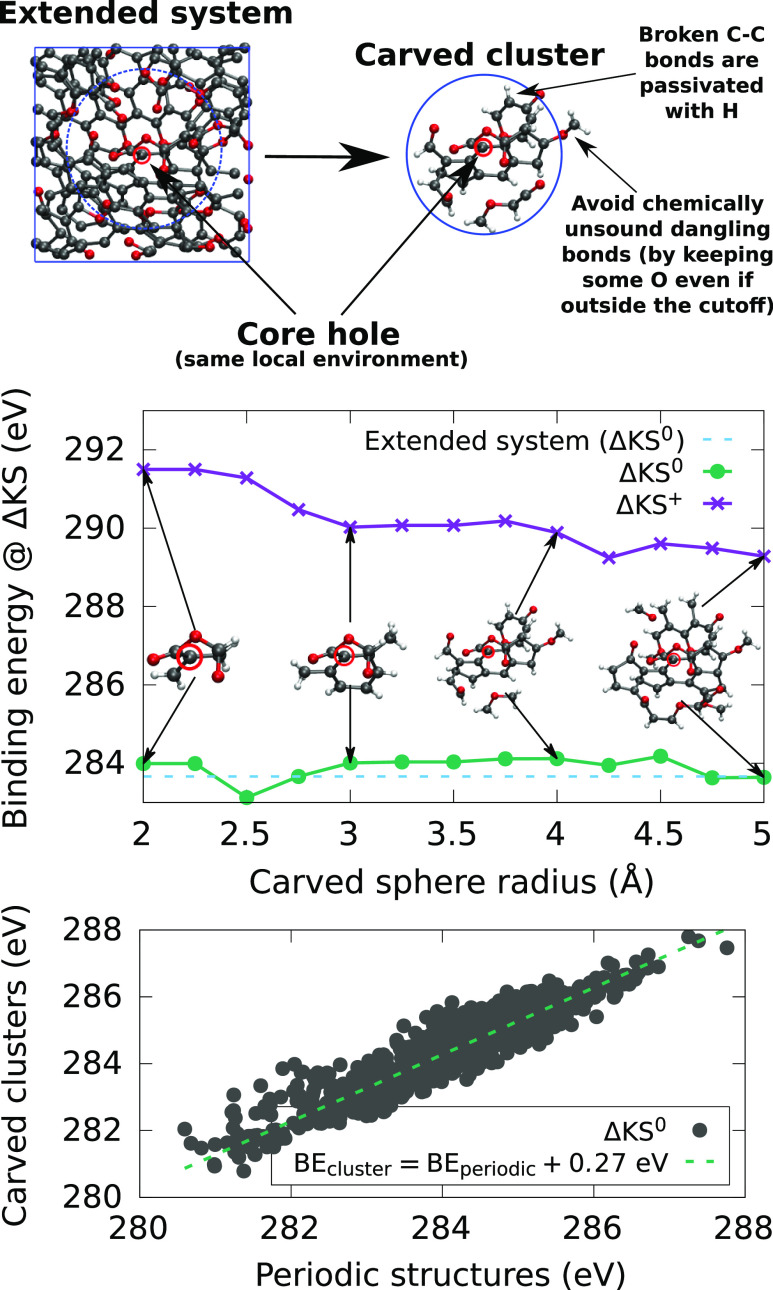
(Top) Example of the cluster carving procedure for an a-CO_*x*_ structure, where the cluster is contained within a sphere centered on the carbon atom highlighted with the red circle where the core hole is created. (Middle) Dependence of the core–electron BE of the cluster on the cutoff radius, together with the periodic reference, for the example structure in the top panel (ΔKS values). (Bottom) Comparison for the whole set of ΔKS data points in our database for which both cluster (*r*_cut_ = 4.25 Å) and periodic ΔKS^0^ values are available, showing that the main difference is simply a small vertical shift in the energies. This strongly indicates that the carved structures are good surrogate models for the extended (periodic) systems. ΔKS^+^ refers to calculations where the excited core electron is removed from the system, whereas in ΔKS^0^ it is promoted to the conduction band, see section 2.2.1. The root-mean-square and mean-absolute errors, once the rigid shift is taken into account, are 0.41 and 0.32 eV, respectively, with a maximum error of 1.69 eV.

The cluster models need to be constructed with care, since creation of dangling bonds or radicals at the cluster surface can affect the overall spin state of the system. We use a “carving” technique introduced in refs (43 and 55), where a spherical portion of the material centered on a specific site is carved out of the bulk material. Broken C–C bonds are passivated with H atoms. All other bonds (C–O, O–H, and C–H) are preserved. The procedure is exemplified in Figure 2, and our carving code is freely available online.^56^

We find that the core-level BEs converge quickly with respect to the cluster radius for ΔKS, as shown in Figure 2 (middle) and also for *GW*, see Figure S1 in the Supporting Information. Convergence is typically reached for *r*_cut_ = 4.25 Å, which is the cluster radius we use for the data acquisition of our ML models. Figure 2 (bottom) also indicates that the carved structures represent a good surrogate model for the periodic structures, since the respective predicted core–electron binding energies closely follow each other. The main difference is a constant shift of 0.27 eV, which is easy to correct for. We attribute the systematic 0.27 eV upward shift to finite-size (or particle-in-a-box) effects upon promotion of the core electron to the conduction band minimum. Since the electron localization length is necessarily reduced in the 4.25 Å cluster compared to the extended structure, the energy of the excited state increases accordingly, by 0.27 eV on average in this case. The validity of the cluster approach is further discussed in section 3.3.

### 2.2 DFT and *GW* Calculations

#### 2.2.1 DFT

We carry out the DFT calculation of core–electron BEs using the ΔKS^16^ total energy method. ΔKS is computationally more affordable and therefore more amenable to high-throughput calculations than its similarly accurate all-electron variant, the ΔSCF^15^ method (see the Supporting Information for an explanation regarding the difference between the ΔKS and ΔSCF methods). In ΔKS, the core-level BE is given as the difference between core-excited and ground-state total energies. In the excited-state calculation, the C 1s or O 1s electron is removed from the core, which is modeled via a special projector augmented-wave (PAW^57^) potential, and only the valence electrons are relaxed. These valence electrons can be relaxed either in the presence of the excited electron (neutral calculation, ΔKS^0^) or in its absence (charged calculation, ΔKS^+^). For molecules, the ΔKS^+^ approximation can be applied directly since the vacuum level is well-defined. For materials, the ΔKS^0^ calculation allows one to align the computed BEs similarly as in experiment, i.e., with respect to the Fermi level. In addition to the periodic ΔKS^0^ calculations, which we need for our ML model, we carried out ΔKS^0^ calculations also for carved clusters to validate that these clusters are indeed good surrogate models for the periodic structures, as shown in Figure 2 (bottom). However, the ΔKS^+^ values are those directly comparable to our *GW* cluster calculations.

We performed open-shell DFT calculations with VASP^58−60^ using the Perdew–Burke–Ernzerhof (PBE) functional.^61^ See the Supporting Information for a discussion on the choice of functional. We apply a constant correction based on GPAW^16,62^ results to convert the relative ΔKS values (i.e., the chemical shifts) from VASP to absolute ΔKS values. Disordered carbon materials often exhibit local atomic magnetization,^11^ which makes the determination of the ground state challenging. We expand on the procedure how to determine the lowest energy magnetic configuration, details of the DFT calculations, and the definition of the reference level for comparison to the experiment in the Supporting Information. Further discussions of energy referencing can also be found in section 3.4 and in ref (13).

#### 2.2.2 GW

The *GW* approximation^29^ is a highly accurate electronic-structure method that can be applied to predict photoemission spectra. The central object of *GW* is the self-energy Σ, which is computed from the Green’s function *G* and the screened Coulomb interaction *W*, where Σ = i*GW*, hence the name *GW*. The self-energy contains all quantum mechanical correlation and exchange interactions between the electrons and the hole created upon photoemission. *GW* offers access to quasiparticle energies, which directly correspond to the negative of the vertical ionization potentials.

*GW* has become the gold standard for the computation of band structures of solids and is now also increasingly applied to molecular valence excitations.^30^ Recently, we advanced the *GW* methodology and implementation for application to deep core excitations by combining exact numeric algorithms in the real frequency domain^28^ with partial self-consistency^63^ and relativistic corrections.^64^ We showed that *GW* reproduces absolute molecular 1s excitations within 0.3 eV of experiment and relative binding energies with average deviations below 0.2 eV.^63^ Our core-level *GW* approach was recently also applied to simple solids,^74^ yielding first promising results. In addition, its extension to the Bethe–Salpeter equation (BSE@*GW*) was lately also successfully used for the prediction of molecular *K*-edge transition energies.^65^

*GW* calculations are several orders of magnitude more expensive computationally than DFT calculations with GGA and even hybrid functionals. Nevertheless, *GW* is nowadays routinely applied to predict valence excitations of systems with several hundred atoms.^66−71^ However, the application of *GW* to deep core excitations is computationally more expensive than for valence states. First, core-level *GW* calculations require more advanced numerical schemes,^28^ increasing the conventional scaling with respect to system size *N* from *O*(*N*^4^) (valence states) to *O*(*N*^5^) (core states). This unfavorable scaling restricts the accessible system size in core-level *GW* to around 100 atoms. Second, an all-electron treatment is necessary, which we efficiently realize by an implementation with localized basis sets. The implementation of *GW* in localized basis set codes is a rather recent development of the past decade,^30^ for which the efficient implementation of periodic boundary conditions is still the subject of ongoing work.^72−74^ Our core-level *GW* implementation^28^ is thus currently restricted to cluster calculations. The largest *GW* calculation in this work was performed for an a-C cluster with 112 atoms on more than 8000 CPU cores.

For the *GW* calculations, we use the FHI-aims program package^75,76^ and follow the procedure developed in ref (63). We employ a single-shot *G*_0_*W*_0_ approach in combination with the PBEh(α = 0.45) functional for the underlying DFT calculation, where α is the amount of exact Hartree–Fock exchange.^77^ The α value was tuned to reproduce the results of computationally more demanding eigenvalue-self-consistent *GW* methods.^63^ We performed a screening for the lowest-energy configuration at the PBEh(α) level to ensure that the open-shell *G*_0_*W*_0_ calculations are performed on top of the DFT ground state. All *GW* results are extrapolated to the complete basis set limit, and relativistic corrections^64^ are added for the O 1s excitations. Further details are given in the Supporting Information. To support open data-driven materials science,^78^ we uploaded the input and output files of all *GW* calculations of the a-C clusters to the Novel Materials Discovery (NOMAD) repository.^79^

### 2.3 Machine-Learning Model

We develop ML models for the prediction of either a core-level BE for atom *i*, BE_*i*_, or the difference Δ_*i*_ between *GW* and DFT predicted core-level BEs, Δ_*i*_ = BE_*i*_^*GW*^ – BE_*i*_^DFT^. Since all our ML models use the same architecture, we denote the quantity to be learned generically as γ_*i*_. Separate models are trained for C 1s and O 1s, which is motivated by the structure of our data: core-level BEs are strongly species dependent and separated by more than 100 eV for different atomic species. C 1s excitations occur around 290 eV, whereas O 1s photoelectrons are ejected at approximately 540 eV. The chemical shifts due to different local atomic environments around a carbon or oxygen core are 2–3 orders of magnitude smaller.

Our ML model is based on kernel ridge regression (KRR), using kernels constructed from soap_turbo descriptors, which are a modification^51^ of the SOAP many-body atomic descriptor,^49^ providing improved speed and accuracy. Here, we used the Python interface (“Quippy”) to the soap_turbo library provided by the QUIP and GAP codes.^80,81^ Briefly, KRR replaces the nonlinear problem of expressing γ_*i*_ for the core of atom *i* as a function of atomic positions, γ_*i*_ = γ({**r**_*j*_ ∈ *S*_*i*_}), where *j* runs through all atoms within an environment of *i*, *S*_*i*_, with a linear problem. Using the “kernel trick”, the same quantity, γ_*i*_, is expressed as a linear combination of kernel functions, *k*(*i*, *t*):
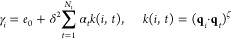
1Here, **q**_*i*_ are the SOAP-type many-body atomic descriptors that we use to encode the atomic information about the environment of atom *i*, and *t* denotes a number of reference environments in the training set. The dot-product SOAP kernel  (where ζ = 2 in our case) provides a measure of similarity between *i* and *t* that is rotationally and translationally invariant.^49^ δ is a parameter, given in eV, which controls the energy scale, and *e*_0_ is a constant reference energy subtracted during training and then added during prediction. Conceptually and methodologically, the present approach is similar to that of the Gaussian approximation potential (GAP) formalism^80,82^ and to our previous models of adsorption energetics in carbon-based materials.^41^ The SOAP descriptors encode atomic structural information up to a certain cutoff radius from the central atom *i*. Thus, we implicitly make the assumption of locality for the binding energies. That is, we assume that only the arrangement of atoms in the immediate vicinity of atom *i* affects the core levels of that atom. The validity of this assumption is illustrated in Figure 2 (middle), where we show that the core-level BE of the central C atom quickly converges with the cluster size. Further confirmation is obtained from Figure 2 (bottom), which shows that the difference between the C 1s excitation from periodic and cluster models is mainly a constant shift.

Model training consists essentially in the inversion of eq 1 (or, more precisely, on a least-squares based optimization of the α_*t*_) for a set of reference calculations, i.e., during training both *i* and *t* run over the same set of atomic environments. To prevent overfitting, we use regularization. Since our data sets for the CHO materials have very few entries (most notably our *GW* data set), to collect error statistics we test all of our models using *n*-fold cross validation, where *n* models are trained, each time leaving out one of the data points, and the model is tested on the particular entry that is left out. For the models based on QM9 data, for which many more training points are available, we train 10 different models for a given training set size, randomizing each time over training configurations and test on the remaining configurations. We do not perform explicit hyperparameter optimization.

The basic ML model architecture used throughout this work is given by eq 1. The application of this model is straightforward for learning the *GW* and DFT predicted molecular C 1s and O 1s BEs of our QM9 subset. For the latter, we have a large amount of *GW* and DFT data, for which we can even train models based on the *GW* data alone. In addition, the data sets are “coherent” in the sense that the DFT and *GW* data sets are of equal size and that the computational data in both sets are well-defined for isolated structures.

However, model training and utilization become more intricate for the CHO materials, where we have few *GW* data, which are in addition only available for the carved clusters. Our main objective for the CHO materials is to combine DFT and *GW* data to (i) improve upon the accuracy of DFT and (ii) overcome the current limitation of *GW* calculations to nonperiodic systems. This implies that we must combine two or more data sets and potentially also two or more ML models. We propose to compute a corrected binding energy (BE^c^) for atom *i* as
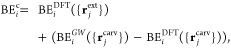
2where {**r**_*j*_^ext^} denotes the atomic environment of *i* within a periodic DFT calculation of the extended structures and {**r**_*j*_^carv^} denotes a truncated representation of this environment, i.e., the one given by a carved cluster centered on *i*. We have therefore split the input data in eq 2 into two terms, a baseline given by  and a correction given by .

The rationale for using eq 2 is the following. First, the Fermi level alignment (important in experimental solid-state XPS because the sample and detector are shorted) is provided by a neutral ΔKS calculation of the extended structure. In this neutral calculation, the BE is computed for the transition of an electron from the core level to the Fermi level; this is the type of ΔKS calculation usually performed for solid-state samples.^13,14,16,24^ Second, the correction to this ΔKS BE, that is, the difference between a *GW* and a ΔKS calculation performed on exactly the same system, is assumed to be (i) local (justifying the use of carved structures) and (ii) independent of whether the core electron is excited to the vacuum level or the Fermi level. While arguably intuitive, there is no formal reason, *a priori*, why these assumptions should hold true. Instead, we verify their validity from the agreement between computational and experimental spectra reported in section 3.

With the data partition in eq 2, we can choose two different routes for predicting BE_*i*_^c^, either (i) train an ML model from {*b*_*i*_ + Δ_*i*_} or (ii) train an ML model from {*b*_*i*_} and another from {Δ_*i*_}, then obtain BE_*i*_^c^ as the sum of both predictions. It is not straightforward to determine *a priori* which option gives more accurate predictions, since the learning rates and available amount of data points are different for each data set. We explore both strategies for the CHO materials studied in this work. The outlined hybrid ML model architecture, i.e., combining data sets from both periodic and cluster calculations, is further described in section 3.3.

## 3 Results and Discussion

We start with a comparison of the *GW* and DFT predicted excitations for the CHO molecules and cluster models. Next, the performance of the ML models for the molecular excitations is discussed. We proceed with results for the ML models, which are the building blocks for our hybrid ML architecture of the CHO materials. We then demonstrate that the hybrid approach is key to achieve quantitatively accurate XPS predictions of CHO materials for three showcases and introduce our XPS Prediction Server.

### 3.1 Comparison of ΔKS and *GW* Excitations

**Figure 3:**
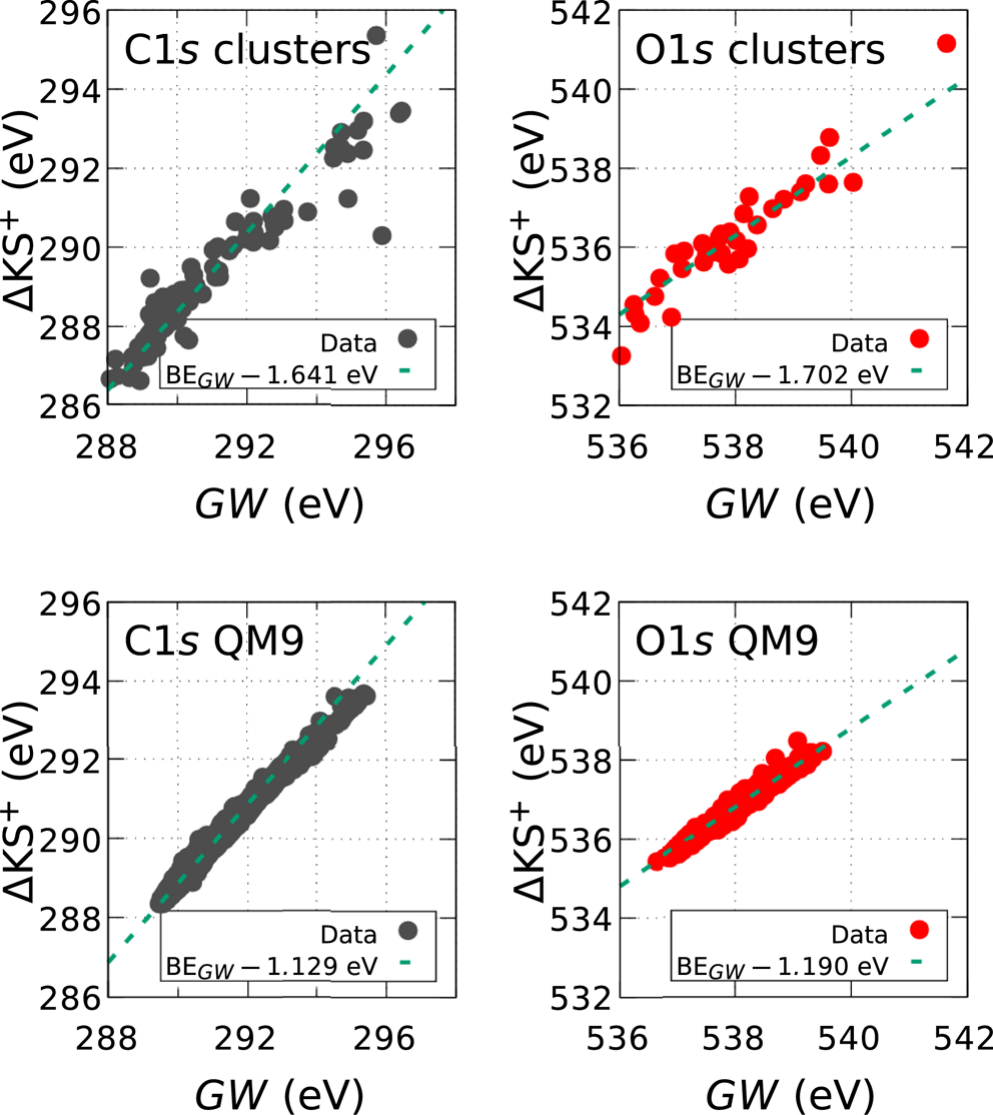
Comparison between the *GW* and ΔKS^+^ results obtained for carved clusters and QM9 molecules. The dashed line indicates a linear fit, where the constant vertical shift gives the leading difference between *GW* and DFT data. This shift is specific to each data set and listed in the legend of each panel.

The results of the ΔKS^+^ and *GW* calculations are shown in Figure 3 and compared to each other for both the cluster models of the solid-state CHO materials and our subset of small CHO-containing molecules from the QM9 data set. Figure 3 allows a direct comparison between the *GW* and DFT predictions, since they are computed on the exact same finite structures and are aligned both at the respective vacuum levels. In all cases depicted in Figure 3, the leading difference between *GW* and DFT BEs, which we can identify as the leading error in the DFT prediction, is a systematic underestimation of the BE. However, this leading error is data set specific. It is in the range of 1.1–1.2 eV for the QM9 subset and around 1.6–1.7 eV for the CHO clusters. In addition, there are some subtle, but important, nonsystematic differences. In the remainder of this section, we illustrate how these subtleties and nonsystematic differences in the data can be absorbed by our ML models as well as how these models can combine data sets to improve the accuracy of the predictions.

### 3.2 Learning Molecular Core–Electron Binding Energies

In the following, we demonstrate how to infer *GW*-quality core-level BEs from a DFT calculation based on our QM9 subset of small CHO molecules. Even though molecular excitations are not the target of this manuscript, the discussion of CHO molecules is instructive because, even at the *GW* level, these systems are small enough that plenty of data can be generated and trends in ML accuracy and learning rates can be closely monitored. We explore three different ways how to avoid an expensive *GW* calculation while retaining *GW* accuracy. (1) A ΔKS^+^ calculation is performed followed by a rigid shift of the obtained BE. (2) An ML model for the difference between *GW* and ΔKS^+^ results is developed, and the ML predicted difference is added to the result of the ΔKS^+^ calculation. (3) An ML model is trained that learns the *GW* data directly. The expected mean absolute errors (MAEs) and root-mean-square errors (RMSE) with respect to the *GW* reference are shown for all three approaches in Table 1 (the method for error estimation is detailed below).

**Table 1:** Expected Errors and Timings When Computing the Core-Electron BE of a CHO-Containing Molecule with Four Different Approaches: (i) Direct *GW* Calculation; (ii) ΔKS Calculation Followed by a Correction Based on a Constant Shift to Account for the Difference between *GW* and ΔKS; (iii) ΔKS Calculation Followed by a Correction Based on an ML Model of the Difference between *GW* and ΔKS; and (iv) Prediction of a ML Model That Learns the *GW* Result Directly^a^

	*GW*	ΔKS^+^+		*GW*_ML_
		shift	(*GW* – ΔKS^+^)_ML_	
C 1s MAE (meV)	0	75	15	27
C 1s RMSE (meV)	0	105	24	38
				
O 1s MAE (meV)	0	77	17	37
O 1s RMSE (meV)	0	95	25	61
				
CPU time (s)	∼300k	∼5k		<1

a The errors are (linearly) extrapolated from the learning curves in Figure 4 to the full size of our CHO-QM9 database: 14 707 and 1 865 unique atomic environments for C and O, respectively. For simplicity, the *GW* error is taken as zero, and the other three approaches are designed to match the *GW* prediction. The CPU time refers to the average computational cost per molecule.

The first approach is motivated by the results in section 3.1, where we found that the leading difference between a *GW* and ΔKS prediction is a constant shift of the energies. If we take a molecule from our QM9 subset and shift the ΔKS^+^ results by +1.129 (C 1s) and +1.190 eV (O 1s), the prediction deviates on average by 75 and 77 meV from the *GW* result, respectively; see also Table 1 for the RMSE. These errors are already quite small. However, this approach still requires a full *ab initio* calculation at the DFT level, which is much cheaper than *GW* but also becomes computationally unfeasible for large disordered carbon structures.

We can improve the speed of the prediction and/or improve the accuracy of the prediction by using ML models. The learning curves for BE_*i*_^*GW*^ and the difference  are reported in Figure 4 for the C 1s and O 1s excitations of the CHO-QM9 subset. Displayed are the ML errors (MAE and RSME) dependent on the number of data points used during training. The errors are computed by testing the models on the portion of the entire database not used for training. For completeness, we included also the learning curves for the ΔKS computed BEs in Figure 4. The models for BE_*i*_^*GW*^ and  exhibit similar learning rates with quickly decreasing errors. The MAE is <40 meV for both ML models when extrapolated to the limit of all the available data (training plus testing). These errors are summarized in Table 1. Figure 4 also shows that learning the difference, Δ_*i*_, is easier than directly learning the core-level BEs, as indicated by an extrapolated MAE of <20 meV. Moreover, the ΔML model achieves the same accuracy with 50 data points as the *GW*_ML_ model with 2000 data points. Finally, we note that it is easier to learn the O 1s data than it is to learn C 1s data. This is due to the higher diversity of possible C atomic environments.

**Figure 4:**
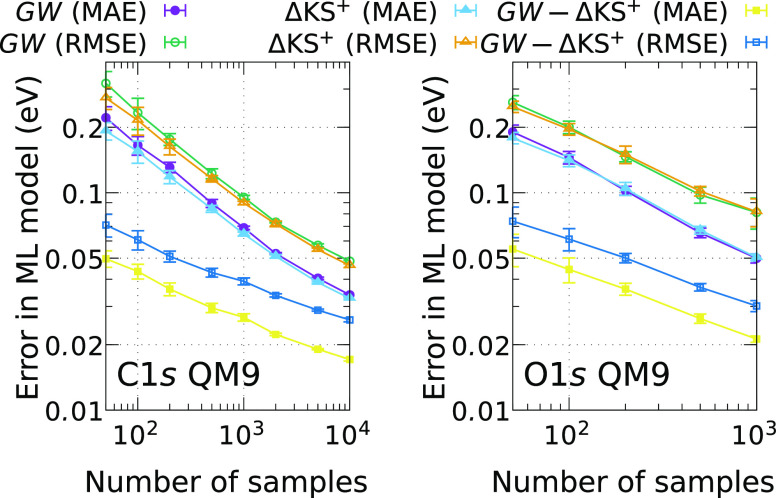
Learning curves for different ML models based on BE data for CHO-containing molecules in the QM9 database. *GW* and ΔKS^+^ models show very similar learning rates, and the ΔML model based on the difference between *GW* and ΔKS^+^ demonstrates an extremely good learning ability. For each training set size *n*, 10 different models are trained, each of which is constructed from *n* randomly chosen training configurations. The errors are then computed by testing the models on all the structures not used for training, i.e., for a given model with *n* training samples, the test set contains 14 707 – *n* and 1865 – *n* samples for C and O, respectively. The error bars are computed by averaging the errors over these 10 different models.

Assuming the *GW* value to be the “golden” standard, the most accurate prediction for CHO molecules is obtained from the ΔKS^+^ + Δ_*i*_^ML^ approach, which reduces the prediction errors to values in the vicinity of 20 meV. Such errors are negligible since they are an order of magnitude smaller than the overall instrumental broadening in XPS experiments and also smaller than the smallest chemical shifts that can be resolved by analyzing experimental XPS spectra (i.e, those that do not overlap). The ML model trained from *GW* data yields the next-best predictions. The worst approach is a ΔKS^+^ calculation followed by a rigid shift, which has the same computational cost as the ΔML-based scheme with an error that is ∼5 times larger; see Table 1. Once the models are trained, the computationally cheapest prediction is obtained from the *GW*_ML_ model, offering the best compromise between accuracy and speed. However, all three strategies are computationally much cheaper than performing an actual *GW* calculation, which is already for small molecules almost 2 orders of magnitude more expensive than a ΔKS calculation (Table 1).

### 3.3 Learning Binding Energies of CHO-Containing Materials

The generation of ΔKS and, in particular, *GW* data is significantly more expensive for CHO materials than for molecules for the following reasons: (i) The size of configuration space, i.e., the different ways in which CHO atoms can be arranged in space, is much larger. This implies that more data are necessary to train ML models of similar quality. (ii) More atoms per site need to be considered to capture the effect of the chemical environment on the excitation energy. This is true even when employing carved structure models, where the number of atoms per atomic environment can be still of the order of 100–200. If the scaling of the method of choice is *N*, this means a cluster calculation is between ∼5^*N*^ and 10^*N*^ times more expensive than a QM9 molecule calculation, since the cluster will contain 5–10 times more atoms than the largest molecules in the QM9 data set.

Another problem is that the difference in the computational cost between DFT and *GW* increases with growing system size. In fact, the highest scaling steps in *GW* only start to dominate the calculation for structures larger than 30–50 atoms,^28^ and hardly affect the computational cost for the CHO molecules. For example, the computational time for a *GW* calculation of a carved cluster with 96 atoms (out of which 38 are C atoms) is 400 000 CPU hours, whereas the ΔKS calculation takes approximately 22 CPU hours for the same system. Compared to the molecular case (see Table 1), the difference in computational cost between *GW* and DFT increased from a factor of 60 to 20 000.

For CHO materials, our ΔKS and *GW* databases are 1 and 2 orders of magnitude smaller, respectively, than for the CHO-QM9 molecules. However, the analysis of the CHO molecules in section 3.2 reveals the solution to this problem. We have seen that a ΔML model, based on the difference between *GW* and ΔKS predictions, can be trained to high accuracy with less data than directly training a model for the BEs at the ΔKS or *GW* level. This justifies the strategy of developing hybrid ML architectures as outlined in section 2.3. Starting from eq 2, we consider two options: (i) We learn a DFT baseline for the extended (“ext”) structures and apply an ML-predicted correction based on the difference between *GW* and DFT for the carved (“carv”) structures as shown in eq 3. (ii) We learn the ΔKS_ext_^0^ baseline and the Δ term simultaneously as in eq 4:
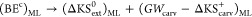
3
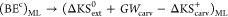
4

**Figure 5:**
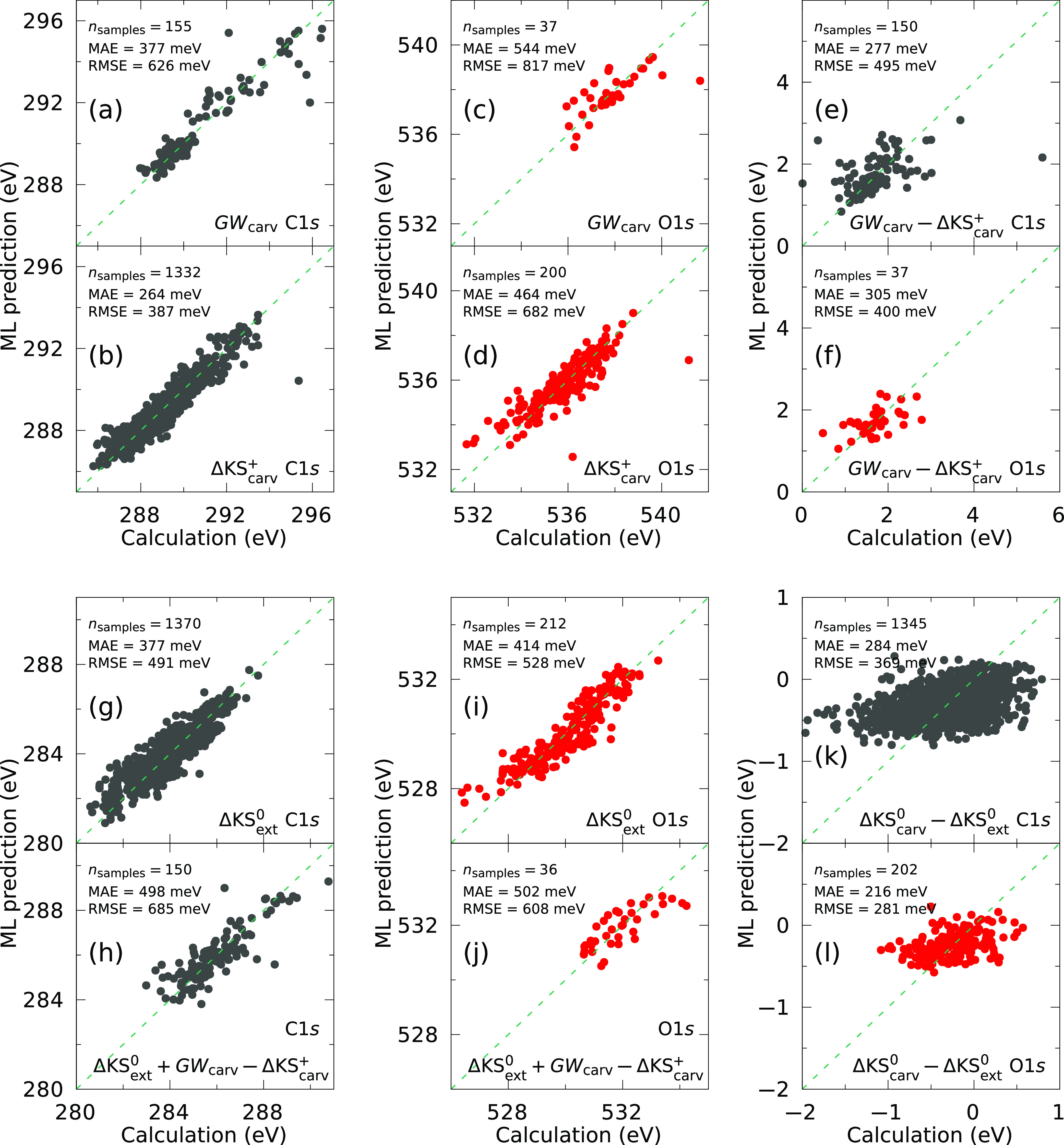
Performance of the different ML models for C 1s (black) and O 1s (red) BEs trained as part of this work. “+” and “0” refer to how the ΔKS simulation is carried out in practice, i.e., by either removing the core electron from the sample or promoting it to the conduction band, respectively. “Ext” stands for “extended” (periodic) structures, as opposed to carved structures (“carv”). See text for a detailed discussion of the figure. The errors were obtained by *n*-fold cross validation, due to the small size of the training sets.

The performance of the ML models required to construct the hybrid ML architectures in eqs 3 and 4 is shown in Figure 5. We start with the discussion of the ingredients for eq 3, i.e., the  models for the BEs of the extended CHO structures (Figure 5g,i) and the ΔML models for the carved structures (Figure 5e,f). For comparison, we also trained ML models for BEs of the carved structures based on *GW* (Figure 5a,c) and ΔKS^+^ data (Figure 5b,d). We observe that the convergence of the *GW* and ΔKS^+^ models for the BEs of the carved structures is much slower than for the molecular case. For instance, the best ΔKS_carv_^+^ model for the C 1s excitations (Figure 5b) still shows a significantly larger error with over 1300 training samples (MAE = 264 meV), when compared to the corresponding CHO-QM9 model in Figure 4 (MAE ∼ 65 meV), corresponding to a 4-fold relative increase of the error. This is easily ascribed to the much more complex configuration space spanned by CHO materials compared to small CHO molecules, as discussed before. Nevertheless, we find that we can train ΔML models (Figure 5e,f) of reasonably good quality (MAE ∼300 meV) for the carved structures with as little as 150 (C 1s) and 37 (O 1s) data points. This is in line with the observation made for molecules that less data are needed for the ΔML models. However, the leading error in eq 3 will originate from the  model (Figure 5g,i) with an MAE of ∼400 meV for both C 1s and O 1s BEs. Its learning behavior is in fact similar to the  model, and the same arguments regarding the complex configuration space apply.

The performance of the ML model where we train the DFT baseline and Δ term at the same time (eq 4), is shown in Figure 5h,j. Despite the small size of our training set (150/36 data points for C 1s/O 1s), we obtain MAEs that are with 500 meV in the range of the overall instrumental broadening in regular XPS experiments (synchrotron-based XPS experiments can achieve better resolution).

The panels k and l in Figure 5 display ΔML models where we compare the core-level BEs from ΔKS^0^ calculations of carved clusters to those of the corresponding periodic structures . The results in Figure 2 (bottom) already indicated that the main difference between core-level BEs of carved and periodic structures is a constant shift. The purpose of training these ΔML models is to assess the validity of the locality assumption inherent to the carving process in more detail (see ref (83) for a general discussion of locality in atomistic modeling and ref (45) for a discussion in the context of GAP force fields). Compared to all other models displayed in Figure 5a–j, we find that the ML models in panels k and l show the poorest *relative* performance, i.e., the largest errors relative to the spread of input values. The MAEs of 284 meV (C 1s) and 216 meV (O 1s) are statistically significant measures for the intrinsic errors due to the carving procedure. These MAEs quantify the influence of the discarded portion of the periodic structure on the core-level BE. In other words, by representing extended structures via carved clusters truncated at 4.25 Å, we will not be able to obtain predictions more accurate than these errors, even in the limit of infinite data. Fortunately, even though *individual* errors from the carving procedure can be expected on the order of 200 meV, the error in the statistical distribution of the predictions is more significant for XPS prediction, since an XPS spectrum is constructed out of the superposition of many individual BE contributions. In addition, we never require ΔKS_carv_ to be an accurate approximation of ΔKS_ext_. Instead, we need the difference between ΔKS_carv_ and *GW*_carv_ to be an accurate approximation of the difference between ΔKS_ext_ and a hypothetical *GW*_ext_ calculation, which we cannot carry out because periodic *GW* core–electron BE calculations are currently unavailable.

Taking the arguments for experimental broadening and statistical distribution into account, we can indeed conclude that the clusters are reasonably good surrogate models for the extended structures, a result that will be corroborated in section 3.4 for actual XPS spectra predictions.

A final observation is that, while it may appear that it is easier to learn C 1s BEs than O 1s BEs, this is solely due to the size of the training sets, which is in turn dictated by the number of available C and O environments in the database. As we saw in Figure 4 for the CHO-QM9 molecules, it is in fact easier to learn O 1s BEs. This is likely due to the higher diversity of possible atomic motifs for carbon^41^ than for oxygen in the CHO system.

### 3.4 Predicting XPS Spectra from the Models

While linking molecular XPS spectra to the computationally predicted BEs from *GW* and ΔKS^+^ is straightforward, this connection is not so clear for materials. There are two main differences that pose significant additional challenges. The first difference is that, in experimental XPS of solid-state samples, the vacuum level is not an easily accessible reference and the experimental BEs are typically reported with respect to the Fermi level of the sample. In the context of electronic structure theory, the Fermi level is only well-defined for metallic systems. For semiconductors and insulators, we need to rely on the thermodynamic definition, in which the Fermi level is given as the derivative of the total (free) energy with respect to the number of electrons in the system. As discussed in section 2.2.1 and the Supporting Information, one possible way to estimate the core-level BE with the Fermi level as reference is to perform a ΔKS^0^ calculation, where we add the excited electron to the conduction band and relax the electronic structure. This is the strategy we follow here and the reason why we learn a DFT baseline at the ΔKS^0^ level in eqs 3 and 4.

The second difference arises precisely from the need for a ΔKS^0^ calculation. The electron that we added to the conduction band will interact with the core hole via the (screened) Coulomb potential leading to a spurious bound exciton. Compared to valence band holes in semiconductors, the core hole is extremely localized and the exciton BE will therefore be quite large (on the order of 0.5 to 1 eV for CHO materials).^84^ Since exciton binding stabilizes the system, the spurious exciton BE lowers the ΔKS prediction, compared to the actual core electron BE, i.e., the one that should be compared to experimental XPS. Dynamical core-hole screening effects are also unaccounted for, which can further complicate direct comparison with experiment. Fortunately, these exciton BEs tend to be highly material-specific and lead to a constant shift of the whole computational XPS spectra (toward lower values). Our future work will aim at quantitative estimation of these exciton BEs for improved core–electron BE prediction. Nevertheless, we find our models to be satisfactorily accurate, even in the absence of excitonic corrections, for the purpose of comparing between computational and experimental spectra. We thus speculate that the contribution of excitonic effects to the chemical shifts in disordered carbon materials may be small enough to not affect this comparison.

The XPS spectra of the CHO materials are computed as the superposition of the individual, experimentally broadened signals of each atom in a given atomic structure (or “supercell”),
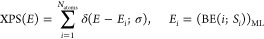
5where *E* is an energy in the spectrum. Each signal *E*_*i*_ is given for atom *i* by an ML model for periodic (“extended”) structures as a function of its atomic environment *S*_*i*_. The latter is characterized via soap_turbo many-body atomic descriptors. The smearing function δ(*E* – *E*_*i*_; σ) is chosen to account for thermal and instrumental broadening. An appropriate choice of broadening function is, e.g., a normalized Gaussian with width σ ≈ 0.5 eV.^14^

We will test three different models for *E*_*i*_ in eq 5, all of which implicitly use the Fermi level as reference. (i) The  model is employed to predict the BEs followed by a rigid shift. The shift is obtained from Figure 3 (top): C 1s excitations are shifted by 1.641 eV and O 1s excitations by 1.702 eV. (ii) The hybrid ML model introduced in eq 3 is used to compute the *GW*-corrected BE^c^. (iii) Equation 4 is employed to obtain an ML prediction for BE^c^. The leading physical assumption for approaches (ii) and (iii) is that the *GW* correction to the charged excitation energies carries over to the neutral excitation case for extended structures. Which of the two *GW*-corrected ML models is optimal strongly depends on the amount of available *GW* data compared to ΔKS data. Even though eq 3 has two sources of error, the error in the first term can be made very small with enough ΔKS_ext_^0^ data. In eq 4, the amount of ΔKS_ext_^0^ data that can be used is limited to those structures for which *GW* data is also available, thus limiting the amount of training data that can be reused.

**Figure 6:**
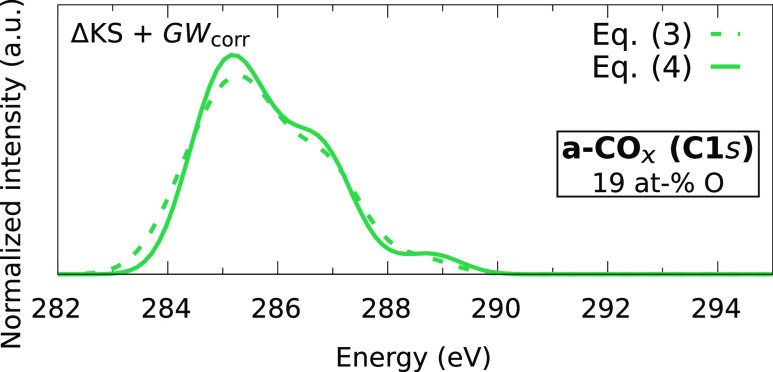
Comparison of the *GW*-corrected ML models for an a-CO_*x*_ sample with 19 at-% O.

For the amount of training data that we managed to gather for this work, both *GW*-corrected ML models perform very similarly. We estimate the RMSE for models based on eqs 3 and 4 to be ∼0.697 and 0.685 eV, respectively, for C 1s predictions, where the error for eq 3 is estimated as the square root of the sum of the individual squared errors (i.e., assuming the individual errors are normally distributed). For O 1s, the estimated RMSEs are 0.662 and 0.608 eV for eqs 3 and 4, respectively. The similar performance manifests also in the prediction of the XPS spectrum of the CHO materials. Figure 6 shows that the predicted peak positions and overall spectrum shape are very similar. For the prediction of the XPS spectrum of selected CHO materials in section 3.5, we will use eq 3 since this ML model has currently more potential to be trained to even higher accuracy by gathering more ΔKS_ext_^0^ data, whereas a performance improvement with eq 4 would also require additional *GW* calculations.

### 3.5 XPS Spectra Predictions for Selected CHO Materials

The ultimate test for the models presented in this paper are predictions of XPS spectra for realistic structural models of CHO materials and subsequent comparison to experiment. We present XPS predictions for three classes of CHO materials: (1) a-C throughout the full range of deposition energies, which in turn covers the full range of sp^2^/sp^3^ ratios observed experimentally; (2) oxygenated amorphous carbon (a-CO_*x*_) with different amounts of oxygen content; and (3) rGO also with varying oxygen concentrations.

**Figure 7:**
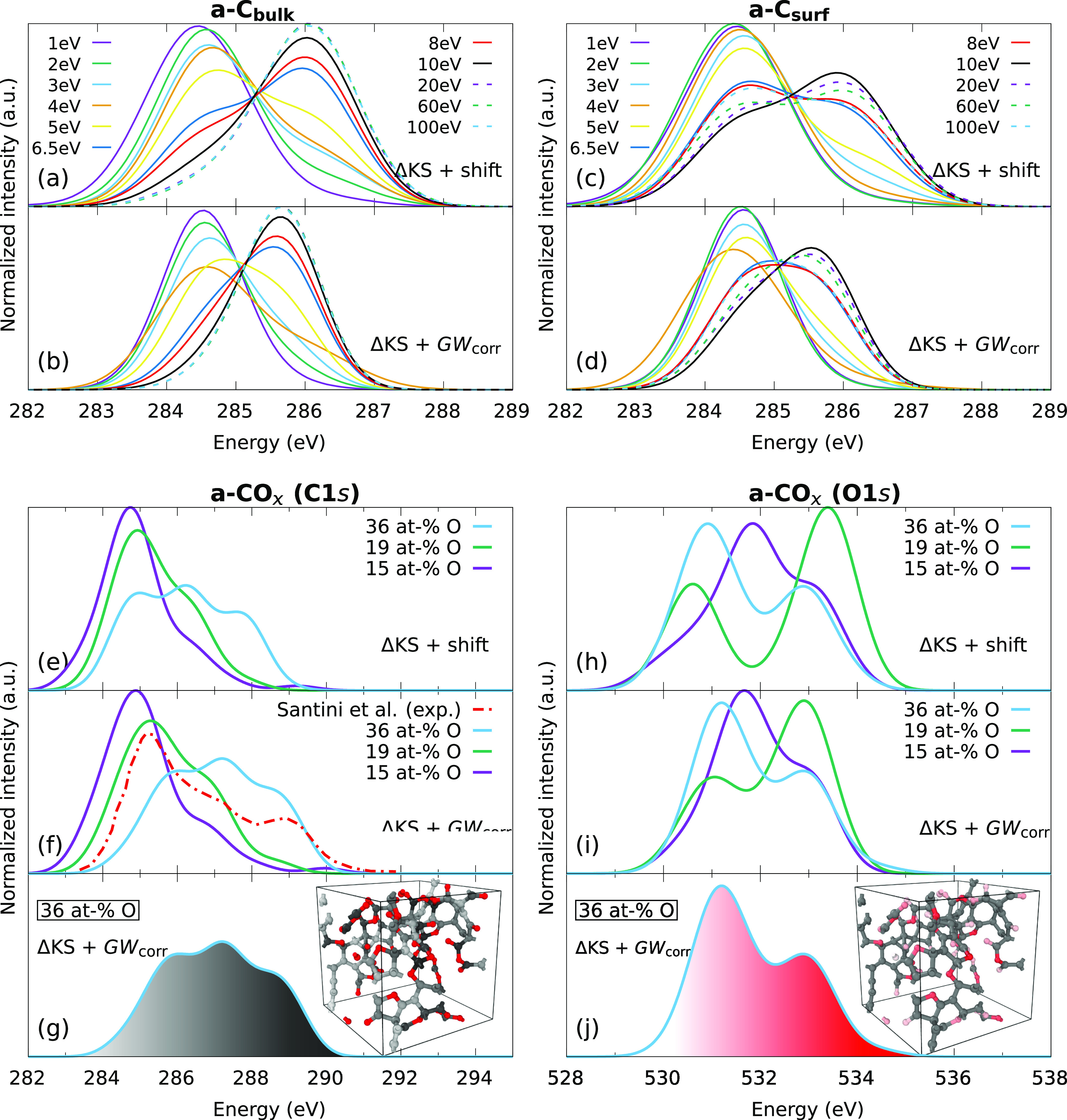
XPS predictions based on our new methodology for C 1s spectra of (a,b) a-C bulk and (c,d) a-C surfaces at different deposition energies and for (e–g) C 1s and (h–j) O 1s spectra of a-CO_*x*_. The panels g and j show site-resolved contributions to the spectra. For example, the light-gray colored C atoms in panel g contribute to the light-gray regions in the spectrum, whereas dark-gray C atoms contribute to the dark-gray spectral regions. The experimental a-CO_*x*_ C 1s data was taken from Santini et al.^8^ Compared are two models:  predictions corrected by (i) a constant shift (+1.641 eV for C 1s and +1.701 eV for O 1s) and (ii) *GW*. All *GW*-corrected predictions in this panel are obtained with eq 3; see the Supporting Information for a comparison of eq 3 and eq 4 for a-CO_*x*_.

Experimentally, a-C thin films are grown by a number of physical deposition methods,^4^ where the main deposition parameter is the kinetic energy of the deposited atoms. Therefore, to model a-C realistically, we use computational structures generated in previous work for deposition energies in the range 1–100 eV.^55,85^ The XPS predictions of the a-C structures are shown in Figure 7a–d, where we have focused on two different regions of thin-film structures: the bulk of the film (panels a and b), on the one hand, and the surface layer (panels c and d), on the other. We present XPS predictions using eq 5 in combination with (i) the  + shift model and (ii) the ML model from eq 3. In the following, we refer to these models as ΔKS + shift and ΔKS + *GW*_corr_, respectively.

The deposition energies control the mass density and sp^2^/sp^3^ content of the a-C films. High deposition energies yield films with high mass densities and sp^3^ contents, whereas low deposition energies correspond to low mass densities and high sp^2^ content.^4,55^ This is also apparent from Figure 7b,d, where we observe a pronounced transition between an sp^2^-dominated XPS spectrum (peak at ≈284.5 eV) for low deposition energy and an sp^3^-dominated (peak at ≈285.6 eV) spectrum at higher deposition energy. The turning point for the transition is between 5 and 6.5 eV incident atom energy. For the bulk, we have a clear transition and the sp^3^ peak at high deposition energies has the same intensity as the sp^2^ peak at low deposition energies. The transition is not fully developed for the surface layer, where the intensity of the sp^3^ peak is less pronounced. The reason is that the surface of the a-C film generally contains lower coordinated atoms compared to the bulk. a-C films are sp^2^-rich at the surface even for very high densities and may contain significant numbers of undercoordinated sp C motifs, which are present only in negligible amounts in the bulk.^55,85^

Comparing the ΔKS + shift and the ΔKS + *GW*_corr_ spectra in Figure 7a–d, we find that the main effect of the *GW* correction is to reduce the width of the predictions. The ΔKS + shift model predicts the sp^3^ peak to be located 0.5 eV higher than the *GW*-corrected model. Experimentally, the separation between sp^2^ and sp^3^ features in a-C has been determined to be of the order of 1.1 eV.^86^ This is the same separation predicted by our *GW*-corrected model, whereas the ΔKS model predicts a separation of ∼1.5 eV. The relative shifts from DFT-based Δ-methods typically agree well with experiment for small molecules.^17^ However, this result indicates that the accuracy of the relative shifts deteriorates for larger systems, which is a consequence of the delocalization error in DFT, demonstrating the need for the *GW* correction.

We discuss next the XPS predictions for a-CO_*x*_ with different oxygen contents (15, 19, and 36 at-% O). Figure 7 shows the C 1s (parts e–g) and O 1s (parts h–j) excitations employing the ΔKS + shift and ΔKS + *GW*_corr_ model from eq 3. The main effect of the *GW* correction is again the reduction of the spread of the predictions, especially for the O 1s spectrum. For a comparison between the ΔKS + *GW*_corr_ models from eq 3 and eq 4, see Figure 6 and Figure S3 in the Supporting Information. The peak alignments are very similar between the two *GW* correction schemes, with slight differences regarding the relative intensity and spread of the lower lying peaks.

The correspondence between excitation energy and atomic motifs is highlighted in panels g and j of Figure 7 with color codings: light-gray (dark-gray) colored C atoms in panel g contribute to light-gray (dark-gray) regions in the C 1s spectrum and light-red (dark-red) colored O atoms in panel j to light-red (dark-red) regions in the O 1s spectrum. For C 1s spectra, the lower energy contributions correspond to carbon–carbon bonds, followed by an increase in the BE as the number of neighboring O atoms increases. The XPS spectra for a-CO_*x*_ materials with higher oxygen content have consequently more features at higher energies since the number of epoxide and ether (C–O–C), keto (C=O), and ester (R–COO–R′) groups increases. The core-level BEs of the C atoms in these groups increase also in that order, where the largest C 1s excitation energies at around 289–290 eV are observed for carboxyl C atoms. For the O 1s spectra, the distribution is essentially bimodal. At lower energies, we observe a peak corresponding to carbonyl O atoms from keto or ester groups. The peak at higher energies originates from contributions of the O atoms in epoxides and ethers and the hydroxyl (singly bound) O atom in the ester groups. The relative intensity of these peaks strongly depends on the oxygen content. In our computational samples, epoxides, ketos, and esters are present in approximately 13:61:26, 42:40:18, and 8:60:32 percentage ratios at 15, 19, and 36 at-% O, respectively.

A comparison to experimental a-CO_*x*_ data is available for the C 1s spectrum from Santini et al.^8^ We observe good agreement for the relative position of the different peaks present in the C 1s spectra with our ΔKS + *GW*_corr_ prediction. The agreement is less good for the peak intensities, but the likely reason is that the relative concentrations of functional groups in the computational and experimental samples are different. We can infer from this direct comparison an oxygen content somewhere in between 19 and 36 at-% (Santini et al. report ≈37% for this sample) and suggest that a combination of those two simulated curves would lead to better agreement with experiment. This in turn suggests that the experimental sample may be inhomogeneous with respect to the oxygen content distribution. Reproducing the experimental structure more closely would require deposition simulations similar to (but more complex than) those in refs (55 and 85), which are nontrivial and beyond the scope of this work. In any case, determining the precise atomic percentages experimentally is difficult because there are instrumental issues (such as calibration), sample issues (heterogeneity, surface roughness), methodological issues (e.g., regarding how the peaks are fitted or how the background was subtracted) and many more.^87−89^ Experimental XPS-derived compositions will also often disagree with other methods, such as X-ray absorption spectroscopy (XAS) or elastic-recoil detection analysis (ERDA), because of sample inhomogeneity and different accessible depths. We show below for rGO that, when more candidate computational structures are available, the atomic percentages can be resolved more precisely by matching predicted and experimentally measured spectra. Therefore, the ability of ML-based XPS predictions to accurately quantify atomic percentages in CHO materials may prove very useful in guiding and interpretation of experiments.

With our third application, rGO, we demonstrate how our developed methodology can be used to assess the validity of candidate structural models for materials. The rGO structures in our database were taken from Kumar et al.^42^ and contain different amounts of oxygen in the range from 10 to 20 at-%. They were either generated from COOH-rich GO (series 1) or OH-rich GO (series 2) precursor structures. Altogether, there are 240 rGO structures with approximately 210 atoms each. We computed the XPS spectra of all of them using the *GW*-corrected ML model of eq 3. Note that, altogether, these ML predictions take only a few minutes on a desktop computer.

**Figure 8:**
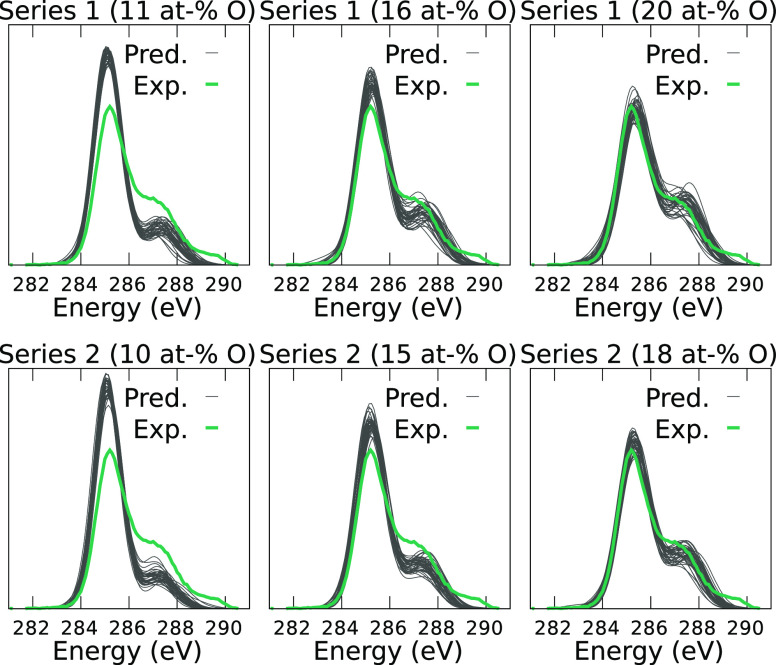
Comparison of C 1s spectra between the computational predictions made with the present XPS model (with *GW* corrections, eq 3) for the rGO structural models of ref (42) and an experimental rGO XPS spectrum from ref (90). “Series 1” and “Series 2” indicate that the computational rGO models were derived from different initial compositions of the GO precursor; series 1 was generated from COOH-rich GO and series 2 from OH-rich GO.^42^ Note that the final content of COOH in rGO for these models is negligible, regardless of the starting configuration used.

In Figure 8, we compare the predicted spectra for series 1 (top) and series 2 (bottom) to the experimental rGO spectrum from ref (90). From Figure 8, we can identify the candidate model structure whose XPS spectrum best matches the experimental one. The spectra of candidate structures with a low oxygen content of 10 or 11 at-% clearly differ from experiment, while the ones with high-O content of 15–20 at-% agree best with the experimental XPS. Clearly, the experimental sample must contain a large fraction of oxygen. However, it is also evident that the structural models with high oxygen content are missing the functional groups that contribute to the feature at ∼289.5 eV in the experimental spectrum. As we saw for the a-CO_*x*_ example, this feature corresponds to carboxyl C atoms. These specific groups are not present in the rGO reference database of Kumar et al.,^42^ even though the rGO structures from series 1 were generated from COOH-rich GO starting configurations. However, unlike hydroxyl groups, the COOH groups are thermodynamically unstable in the computational structure generation process and are not present in the final structural models. Our analysis thus indicates that the composition of the experimental sample is similar, but not identical, to the structural models with high O-content. In particular, it sheds light onto the missing bits of information, in this case, the presence of COOH groups.

### 3.6 The XPS Prediction Server

We have set up an online tool that utilizes the different ML models described throughout this paper. The XPS Prediction Server is available for free at nanocarbon.fi/xps. The user can upload a model structure in any format readable by the Atomic Simulation Environment (ASE),^91^ and the server will execute a Python script that runs the descriptor construction (via calls to Quippy^81^) and performs the kernel regression according to a model of choice. At the moment, only the CHO models described herein are available, but models for other materials can be uploaded in the future as they are developed. The tool works in a fully automated way, and for systems of usual sizes in the context of DFT modeling of materials (a couple hundreds of atoms), a prediction can be obtained within seconds. It is our hope to extend this concept of ML-based computational prediction to other materials and experimental observables, most notably other spectroscopic techniques.

## 4 Summary and Outlook

We have presented an ML-based methodology to predict quantitatively accurate XPS spectra for CHO-containing molecules and materials. We generated a comprehensive database of computational core-level BEs from DFT and *GW* calculations. By careful combination of DFT and *GW* data, accurate ML models were trained for C 1s and O 1s excitations from relatively small data sets. For molecular BEs, we showed that the errors in the ML predictions can be reduced to less than 50 meV. The ML models were then applied to generate XPS spectra of selected CHO materials, namely, a-C thin films, a-CO_*x*_, and rGO with different oxygen concentrations. Our predictions show excellent qualitative and quantitative agreement with experiment, resolving spectral shapes and features within 0.1 eV for the selected disordered carbon-based materials. We also showed that ML models trained with DFT data alone cannot reach this level of predictive power and that data from the more accurate *GW* approach are indeed crucial. For disordered materials, we expect that whenever a suitably constructed database with more *GW* data is available, an XPS ML model can be trained to provide accuracy close to the practical resolution of common XPS experimental equipment.

We demonstrated the potential and suitability of our computational XPS tool to, e.g., quantify the atomic percentages in a-CO_*x*_ or identify shortcomings in candidate structure models of rGO. We have made this new methodology freely available to the public through the XPS Prediction Server. Such a computational tool may prove valuable in guiding and interpreting experimental work and in validating computational structural models of materials. We hope to extend our ML models to other material classes and spectroscopic techniques (XAS, Raman, IR, NMR, etc.) in the future.

## Supporting Information Available

Further details pertaining the calculations presented in this paper, in particular technical information about the *GW* and ΔKS calculations; *GW* convergence studies for the C 1s energies with respect to the cluster radius; an MDS map of CHO structures using a larger cutoff; and a repeat of the XPS spectra predictions using a different ML model (PDF)
