# Supplementary material for: Accurate Computational Prediction of Core-Electron Binding Energies in Carbon-Based Materials: A Machine-Learning Model Combining Density-Functional Theory and GW
Source: Chem Mater. 2022 Jul 13;34(14):6240–54. doi: 10.1021/acs.chemmater.1c04279 (PMC9330771; doi:10.1021/acs.chemmater.1c04279)
Supplement: Supplementary file 1 — cm1c04279_si_001.pdf [file cm1c04279_si_001.pdf]

Supporting Information:

Accurate computational prediction of core-electron binding energies in carbon-based materials: A machine-learning model combining density-functional theory and GW

Dorothea Golze,[1,2,*] Markus Hirvensalo,[2] Patricia Hernández-León,[3] Anja Aarva,[3] Jarkko Etula,[4] Toma Susi,[5] Patrick Rinke,[2] Tomi Laurila,[3,4] and Miguel A. Caro[3,†]

[1]*Faculty of Chemistry and Food Chemistry, Technische Universität Dresden, 01062 Dresden, Germany*
[2]*Department of Applied Physics, Aalto University, 02150, Espoo, Finland*
[3]*Department of Electrical Engineering and Automation, Aalto University, 02150, Espoo, Finland*
[4]*Department of Chemistry and Materials Science, Aalto University, 02150, Espoo, Finland*
[5]*University of Vienna, Faculty of Physics, Boltzmanngasse 5, 1090, Vienna, Austria*

[*] dorothea.golze@tu-dresden.de
[†] mcaroba@gmail.com

I. DISCUSSION OF DFT AND GW PROCEDURE

A. Core-level binding energies from DFT

DFT calculation of core-electron BEs can either rely on eigenvalue differences, which are computationally cheap but inaccurate, or total energy differences, which are significantly more expensive but also much more accurate. Within the total energy methods, at least two stand out: "delta self-consistent field" (ΔSCF) [1] and "delta Kohn-Sham" (ΔKS) [2]. Both methods compute the core-level BE as the difference between core-excited and ground-state total energies, i.e., two DFT calculations are required to estimate each individual core-electron BE [3]. Within the context of BE calculations, ΔSCF typically refers to all-electron (full potential) calculations, whereas ΔKS can be considered as its projector augmented-wave (PAW) equivalent. In the excited state calculations of ΔKS and ΔSCF, an electron is removed from the core; in this study from the C1s and O1s levels. In ΔSCF all remaining electrons are allowed to relax, whereas in ΔKS the core is described by a PAW potential including an explicit (spherically symmetric) core hole, and only the valence electrons are relaxed. These two techniques have been compared for carbon materials in Ref. [4], where systematically smaller C1s BEs were reported from ΔKS. Here we employ the ΔKS technique, since it is computationally less demanding than ΔSCF. In addition, ΔKS does not require the application of occupation constraints because the core hole is frozen after its creation in the atomic core. In ΔSCF, occupation constraints are sometimes difficult to enforce and thus not ideal for high-throughput calculations.

After the creation of the core hole, the valence electronic structure is relaxed either in the presence of the excited electron (neutral calculation, ΔKS^0) or in its absence (charged calculation, ΔKS^+), as already described in the main text.

B. Definition of the reference level in extended systems

The ΔKS^+ approximation can be applied to molecules and finite systems, where the vacuum level is well defined. For extended systems, the vacuum level is not an easily accessible reference and the experimental BEs are commonly reported with respect to the Fermi level. In the context of electronic structure calculations, the Fermi level is only well defined for metallic systems. For semiconductors and insulators, we need to rely on the thermodynamic definition, in which the Fermi level is given as the derivative of the total (free) energy with respect to the number of electrons in the system.

In the ΔKS^0 approximation we therefore add the excited electron to the conduction band and relax the electronic structure. A side effect of this approach is that the electron in the conduction band will interact with the core hole via the Coulomb interaction and form an electron-hole pair resembling a bound core-hole exciton. The energy required to break the exciton is approximately equal to the energy required to promote the bound electron to the Fermi level, i.e., the exciton binding energy. Importantly, the need to explicitly pin down the location of the Fermi level in the absolute scale does not arise within this scheme. Unfortunately, it is currently not straightforward to estimate the exciton binding energy within the same methodological framework we use to compute the ΔKS total energy differences. Work to find a computationally affordable way to solve this issue is ongoing. A distinct advantage of the ΔKS^0 approximation is that the electron in the conduction band restores the charge neutrality of the supercell and thus avoids the Coulomb divergence in charged periodic systems.

In GW, the response of the system to a (core) hole is expressed through many-body perturbation theory. The explicit optimization of the wave function of an ionized system is avoided and the method is therefore, unlike ΔKS or ΔSCF, well defined for periodic structures. The reference level problem for absolute core level BEs remains also for GW calculations of solids. Previous work referenced core-level BEs to the valence band maximum [5, 6]. The same referencing convention was recently used for DFT-based Δ-methods [7] and was found to be more appropriate due to the uncertainty in determining the Fermi level in experiment. All in all, the referencing in computational core-level spectroscopy remains far from trivial, but ongoing efforts in the community are helping to establish more robust strategies to address this important issue.

C. Computational details for ΔKS calculations

For all the DFT calculations we use the VASP code [8–10] (tag `ICORELEVEL = 2`) with the PBE functional [11]. We benchmarked the VASP results against GPAW [2, 12], which we have successfully used in previous work for carbon-based systems [4, 13–16]. While both implementations differ somewhat, they yield similar results for most systems. We chose VASP for this work since it shows better DFT self-consistency convergence for open-shell systems; this consideration is important for disordered carbon materials that exhibit local atomic magnetization [17]. Unfortunately, VASP only reports relative ΔKS values (i.e., the "chemical shifts"), whereas GPAW allows us to compute the absolute ΔKS values. By comparing the GPAW and VASP data, it is straightforward to derive the appropriate approximate shifts, which need to be applied to the VASP-reported energies: +337.5 eV and +664.5 eV for C1s and O1s BEs, respectively. Therefore, all ΔKS energies reported in this work have been shifted by these amounts.

D. Choice of the DFT functional for ΔKS

Although meta-GGA functionals like SCAN [18] can yield more accurate core-electron BEs [19–21], our tests with SCAN and r^2SCAN [22, 23] showed similar BE trends, 2 to 3 times as much CPU cost per self-consistent-field (SCF) iteration, and significantly more difficulty in achieving SCF convergence for our complex systems, compared to PBE. Given the large number of DFT calculations performed for this work (circa 40,000 individual calculations), and the fact that the DFT calculations are primarily used as a baseline on top of which GW corrections are applied, the PBE functional was chosen as a robust framework for our high-throughput calculations.

E. Computational details for *GW* calculations

The GW calculations were carried out with the FHI-aims program package [24, 25], where the all-electron KS equations are solved using numeric atom-centered basis functions. In this work, we use a single-shot G_0W_0 approach. For the underlying DFT calculation, we employ the PBEh(α) functional family with an adjustable amount α of Hartree-Fock exact exchange [26]. We showed in our previous work [27], that a value of $\alpha = 0.45$ reproduces the results of computationally more demanding eigenvalue-self-consistent GW methods. In previous work [27, 28] we denoted this scheme G_0W_0@PBEh($\alpha = 0.45$). The contour deformation technique [29–31] is used to evaluate the frequency integral of the self energy, and a modified Gauss-Legendre grid [25] with 200 grid points is employed for the imaginary frequency integral. The quasiparticle equation is solved iteratively. Like other correlated electronic-structure methods, GW converges slowly with respect to basis set size [31–34]. Therefore, we use a similar procedure as in our previous work [27, 28] and extrapolate the core-level BEs to the complete basis set limit by performing a 2-point extrapolation using the Dunning basis sets cc-pVTZ and cc-pVQZ [35, 36]. The extrapolation is performed by a linear regression against the inverse of the total number of basis functions. All virtual states resolved by the respective basis set are included in the G_0W_0 calculations. We performed non-relativistic G_0W_0 calculations and added a relativistic correction [28] of 0.42 eV to the O$1s$ BEs from G_0W_0. We neglected the relativistic correction for C$1s$, which is about 0.12 eV, since the α-value of 0.45 is slightly too large for C$1s$ excitations and thus already contains the relativistic corrections, see also Figure 3 (a,b) in Ref. 28 for details on the α-tuning.

F. Determination of the DFT ground state

Determining the ground state for the a-C clusters and extended structures is challenging due to their local atomic magnetization. The lowest energy magnetic configuration for the ΔKS calculations of the clusters and the periodic structures was found by carrying out seven different VASP calculations at the PBE level fixing the total magnetic moment between 0 and 6 with an increment of 1 and then choosing the lowest energy one. The G_0W_0 calculations start from a DFT run with the PBEh($\alpha = 0.45$) functional, for which the ground state had to be determined as well. Ten DFT calculations with different spin initialization (including minimal and maximal magnetization) were performed at the PBEh($\alpha = 0.45$) level for each structure and basis set. The G_0W_0 calculations were then performed on top of the DFT run with the lowest total energy. Since the ΔKS and G_0W_0 calculations are both open-shell, we obtain BEs for both spin channels, which are typically very close in energy (< 0.5 eV). For learning the ML models, the BEs from both spin channels were averaged.

II. *GW* CONVERGENCE WITH RESPECT TO CLUSTER SIZE

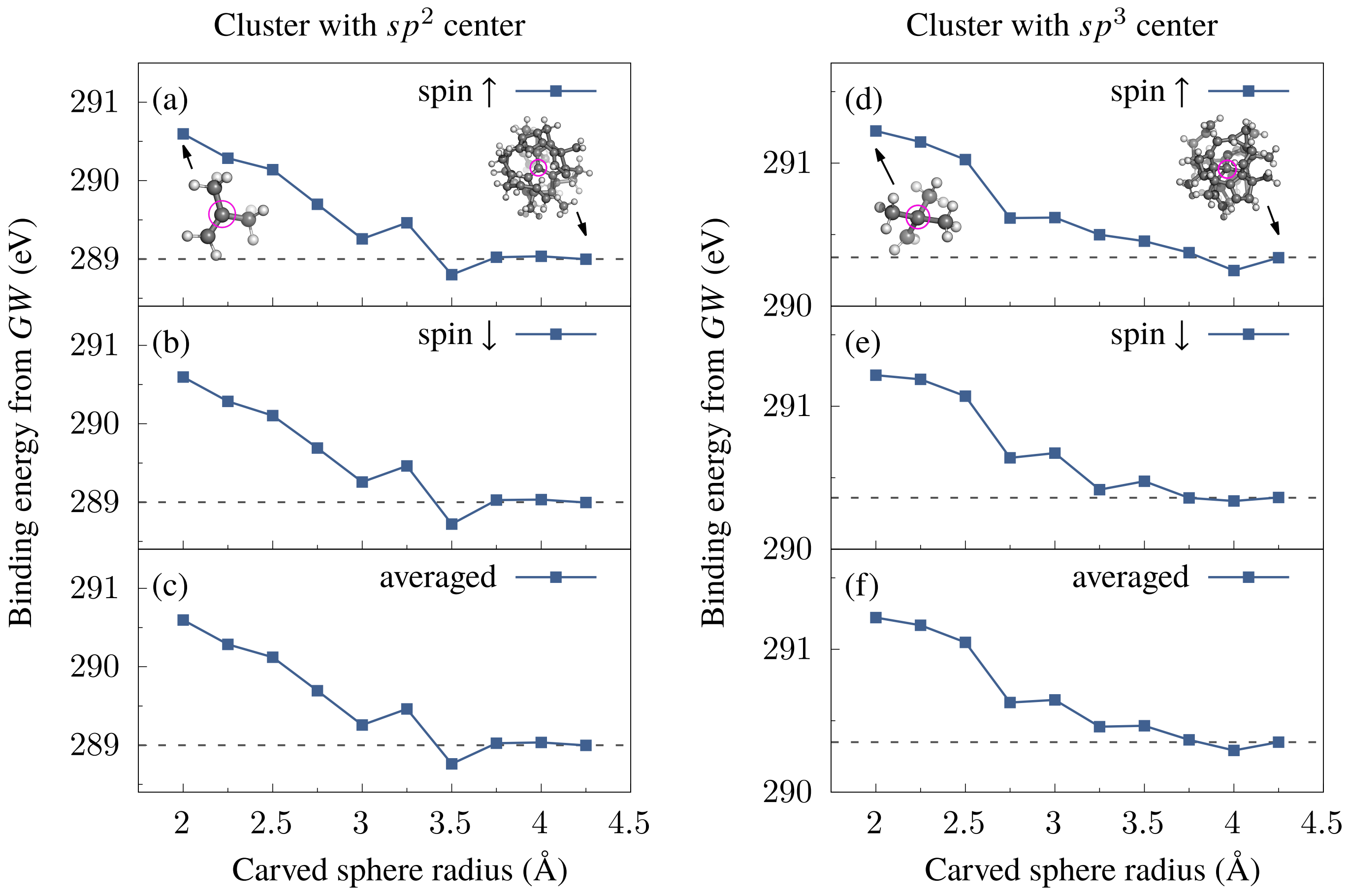

FIG. S1. Convergence of the C1s binding energy (BE) from GW with respect to the cluster size. Displayed are the BEs of the central carbon atom encircled in pink. (a-c) Cluster with an sp^2 central site. (d-f) Cluster with an sp^3 central site. The a-C structures are spin-polarized. BEs for the separate spin channels are shown in (a,b) and (d,e). The BEs averaged over both spin channels are given in (c,f). The dashed line indicates the converged BE. The GW results are extrapolated to the complete basis set limit.

III. ML MODEL FOR NEUTRAL CALCULATION OF CARVED STRUCTURE

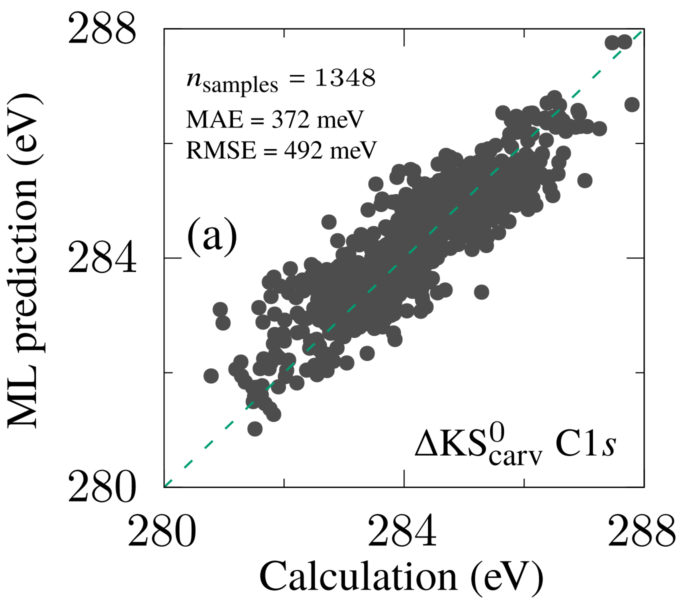

FIG. S2. ML models for (a) C1s and (b) O1s BEs based on ΔKS^0 data for the carved structures.

IV. COMPARISON BETWEEN THE TWO GW-CORRECTED MODELS

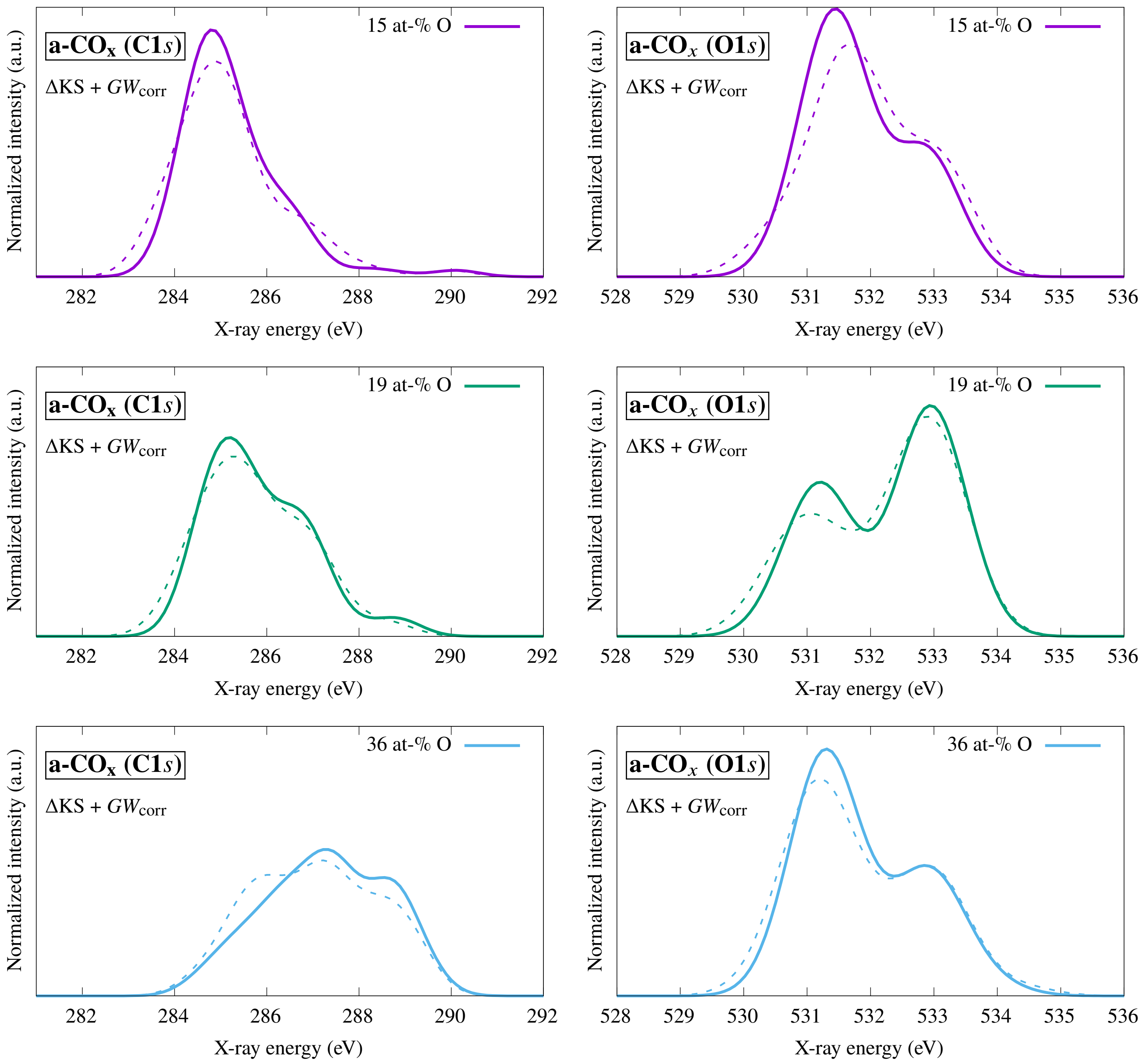

FIG. S3. Comparison for the performance of the two GW-corrected models we have trained for the a-CO_x samples. Solid lines show predictions of the "single model" concept, Eq. (4) of the main paper, and dashed lines show the predictions of the "double model" concept, Eq. (3) of the main paper. The peak positions and overall shapes of the predicted spectra are quite similar, only differing in the fine detail.

V. MULTIDIMENSIONAL SCALING MAP WITH EXTENDED CUTOFF

[1] P. S. Bagus, "Self-consistent-field wave functions for hole states of some Ne-like and Ar-like ions," Phys. Rev. **139**, A619 (1965).

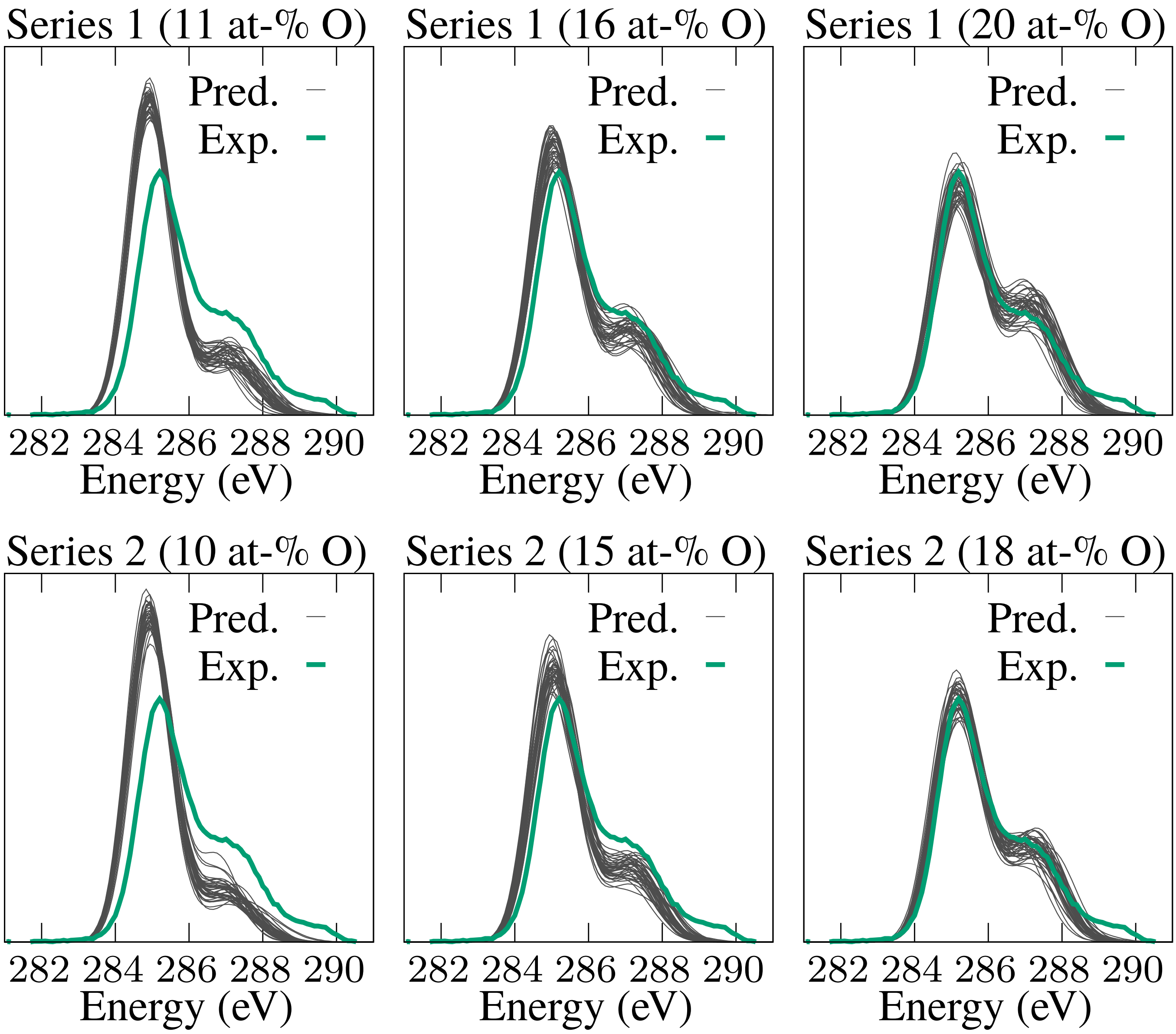

FIG. S4. Reduced graphene oxide calculations, Fig. 8 of the main paper, repeated here with the single model, Eq. (4) of the main paper.

[2] M. P. Ljungberg, J. J. Mortensen, and L. G. M. Pettersson, "An implementation of core level spectroscopies in a real space projector augmented wave density functional theory code," J. Electron Spectrosc. **184**, 427 (2011).

[3] When computing all the BEs for a given structure with N atoms, the ground state calculation only needs to be carried out once per atom, i.e., leading effectively to $N+1$ calculations, rather than $2N$. However, in our case we only compute one BE per structure, since the carved surrogate structure is unique to each specific atom. In this case, the number of require calculations is indeed at least $2N$. Because we also probe different magnetic configurations, the actual number of calculations is even larger.

[4] T. Susi, D. J. Mowbray, M. P. Ljungberg, and P. Ayala, "Calculation of the graphene C $1s$ core level binding energy," Phys. Rev. B **91**, 081401 (2015).

[5] T. Aoki and K. Ohno, "Accurate quasiparticle calculation of x-ray photoelectron spectra of solids," J. Phys.: Condens. Matter **30**, 21LT01 (2018).

[6] T. Zhu and G. K.-L. Chan, "All-electron Gaussian-based G_0W_0 for valence and core excitation energies of periodic systems," J. Chem. Theory Comput. **17**, 727 (2021).

[7] J. M. Kahk and J. Lischner, "Core electron binding energies in solids from periodic all-electron Δ-self-consistent-field calculations," arXiv:2104.06356 (2021).

[8] G. Kresse and J. Furthmüller, "Efficient iterative schemes for *ab initio* total-energy calculations using a plane-wave basis set," Phys. Rev. B **54**, 11169 (1996).

[9] G. Kresse and D. Joubert, "From ultrasoft pseudopotentials to the projector augmented-wave method," Phys. Rev. B **59**, 1758 (1999).

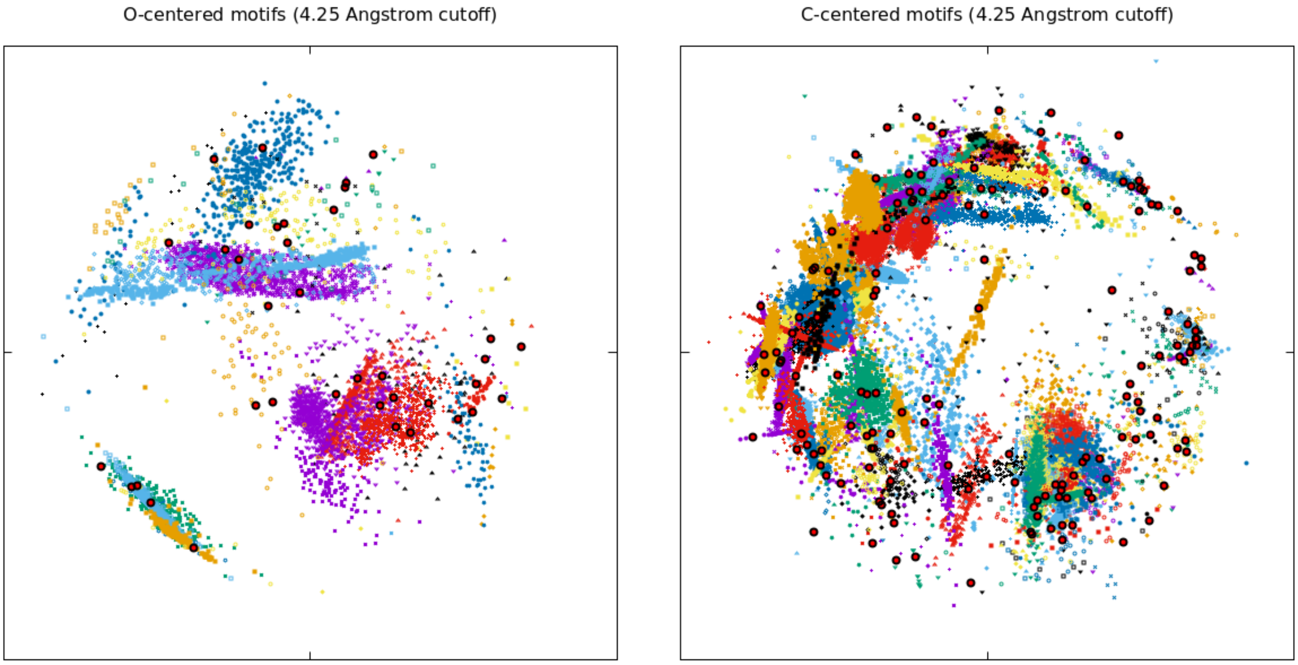

FIG. S5. Multidimensional scaling map with a 4.25 Å cutoff radius. The large red dots with black contour indicate the position of the medoids, which were shown as gray triangles in Fig. 1 of the main manuscript. In this figure we can see how our sparsification strategy selects homogeneously distributed training points in configuration space, rather than overrepresenting the most populated atomic motifs in the database (which appear as dense data clusters on this plot).

[10] L. Köhler and G. Kresse, “Density functional study of CO on Rh(111),” Phys. Rev. B **70**, 165405 (2004).

[11] J. P. Perdew, K. Burke, and M. Ernzerhof, “Generalized gradient approximation made simple,” Phys. Rev. Lett. **77**, 3865 (1996).

[12] J. Enkovaara, C. Rostgaard, J. J. Mortensen, J. Chen, M. Dułak, L. Ferrighi, J. Gavnholt, C. Glinsvad, V. Haikola, H. A. Hansen, H. H Kristoffersen, M. Kuisma, A. H. Larsen, L. Lehtovaara, M. Ljungberg, O. Lopez-Acevedo, P. G. Moses, J. Ojanen, T. Olsen, V. Petzold, N. A. Romero, J. Stausholm-Møller, M. Strange, G. A. Tritsaris, M. Vanin, M. Walter, B. Hammer, H. Häkkinen, G. K. H. Madsen, R. M. Nieminen, J. K. Nørskov, M. Puska, T. T. Rantala, J. Schiøtz, K. S. Thygesen, and K. W. Jacobsen, “Electronic structure calculations with GPAW: a real-space implementation of the projector augmented-wave method,” J. Phys.: Condens. Matter **22**, 253202 (2010).

[13] T. Susi, M. Kaukonen, P. Havu, M. P. Ljungberg, P. Ayala, and E. I. Kauppinen, “Core level binding energies of functionalized and defective graphene,” Beilstein J. Nanotech. **5**, 121 (2014).

[14] S. Sainio, D. Nordlund, M. A. Caro, R. Gandhiraman, J. Koehne, N. Wester, J. Koskinen, M. Meyyappan, and T. Laurila, “Correlation between sp^3-to-sp^2 ratio and surface oxygen functionalities in tetrahedral amorphous carbon (ta-C) thin film electrodes and implications of their electrochemical properties,” J. Phys. Chem. C **120**, 8298 (2016).

[15] A. Aarva, V. L. Deringer, S. Sainio, T. Laurila, and M. A. Caro, “Understanding X-ray spectroscopy of carbonaceous materials by combining experiments, density functional theory and machine learning. Part I: fingerprint spectra,” Chem. Mater. **31**, 9243 (2019).

[16] A. Aarva, V. L. Deringer, S. Sainio, T. Laurila, and M. A. Caro, “Understanding X-ray spectroscopy of carbonaceous materials by combining experiments, density functional theory and machine learning. Part II: quantitative fitting of spectra,” Chem. Mater. **31**, 9256 (2019).

[17] T. Laurila, S. Sainio, and M. A. Caro, “Hybrid carbon based nanomaterials for electrochemical detection of biomolecules,” Prog. Mater. Sci. **88**, 499 (2017).

[18] J. Sun, A. Ruzsinszky, and J. P. Perdew, “Strongly constrained and appropriately normed semilocal density functional,” Phys. Rev. Lett. **115**, 036402 (2015).

[19] N. Pueyo Bellafont, F. Viñes, and F. Illas, “Performance of the TPSS functional on predicting core level binding energies of main group elements containing molecules: A good choice for molecules adsorbed on metal surfaces,” J. Chem. Theory Comput. **12**, 324 (2016).

[20] J. M. Kahk and J. Lischner, "Accurate absolute core-electron binding energies of molecules, solids, and surfaces from first-principles calculations," Phys. Rev. Mater. **3**, 100801 (2019).
[21] D. Hait and M. Head-Gordon, "Highly accurate prediction of core spectra of molecules at density functional theory cost: Attaining sub-electronvolt error from a restricted open-shell kohn–sham approach," J. Phys. Chem. Lett. **11**, 775–786 (2020), pMID: 31917579, https://doi.org/10.1021/acs.jpclett.9b03661.
[22] A. P. Bartók and J. R. Yates, "Regularized SCAN functional," J. Chem. Phys. **150**, 161101 (2019).
[23] J. W. Furness, A. D. Kaplan, J. Ning, J. P. Perdew, and J. Sun, "Accurate and numerically efficient r^2SCAN meta-generalized gradient approximation," J. Phys. Chem. Lett. **11**, 8208 (2020).
[24] V. Blum, R. Gehrke, F. Hanke, P. Havu, V. Havu, X. Ren, K. Reuter, and M. Scheffler, "Ab initio molecular simulations with numeric atom-centered orbitals," Comput. Phys. Commun. **180**, 2175 (2009).
[25] X. Ren, P. Rinke, V. Blum, J. Wieferink, A. Tkatchenko, A. Sanfilippo, K. Reuter, and M. Scheffler, "Resolution-of-identity approach to Hartree-Fock, hybrid density functionals, RPA, MP2 and GW with numeric atom-centered orbital basis functions," New J. Phys. **14**, 053020 (2012).
[26] V. Atalla, M. Yoon, F. Caruso, P. Rinke, and M. Scheffler, "Hybrid density functional theory meets quasiparticle calculations: A consistent electronic structure approach," Phys. Rev. B **88**, 165122 (2013).
[27] D. Golze, L. Keller, and P. Rinke, "Accurate absolute and relative core-level binding energies from gw," J. Phys. Chem. Lett **11**, 1840 (2020).
[28] L. Keller, V. Blum, P. Rinke, and D. Golze, "Relativistic correction scheme for core-level binding energies from gw," J. Chem. Phys. **153**, 114110 (2020).
[29] M. Govoni and G. Galli, "Large scale GW calculations," J. Chem. Theory Comput. **11**, 2680 (2015).
[30] D. Golze, J. Wilhelm, M. J. van Setten, and P. Rinke, "Core-level binding energies from GW: An efficient full-frequency approach within a localized basis," J. Chem. Theory Comput. **14**, 4856 (2018).
[31] D. Golze, M. Dvorak, and P. Rinke, "The GW compendium: A practical guide to theoretical photoemission spectroscopy," Front. Chem. **7**, 377 (2019).
[32] F. Bruneval, "Ionization energy of atoms obtained from GW self-energy or from random phase approximation total energies," J. Chem. Phys. **136**, 194107 (2012).
[33] F. Bruneval and M. A. L. Marques, "Benchmarking the starting points of the GW approximation for molecules," J. Chem. Theory Comput. **9**, 324 (2013).
[34] J. Wilhelm, M. Del Ben, and J. Hutter, "GW in the Gaussian and plane waves scheme with application to linear acenes," J. Chem. Theory Comput. **12**, 3623 (2016).
[35] T. H. Dunning, "Gaussian basis sets for use in correlated molecular calculations. I. The atoms boron through neon and hydrogen," J. Chem. Phys. **90**, 1007 (1989).
[36] A. K. Wilson, T. van Mourik, and T. H. Dunning, "Gaussian basis sets for use in correlated molecular calculations. VI. Sextuple zeta correlation consistent basis sets for boron through neon," J. Mol. Struct. (Theochem) **388**, 339 (1996).
